# Initial characterization of gap phase introduction in every cell cycle of *C. elegans* embryogenesis

**DOI:** 10.3389/fcell.2022.978962

**Published:** 2022-10-25

**Authors:** Ming-Kin Wong, Vincy Wing Sze Ho, Xiaotai Huang, Lu-Yan Chan, Dongying Xie, Runsheng Li, Xiaoliang Ren, Guoye Guan, Yiming Ma, Boyi Hu, Hong Yan, Zhongying Zhao

**Affiliations:** ^1^ Department of Biology, Hong Kong Baptist University, Kowloon, Hong Kong SAR, China; ^2^ School of Computer Science and Technology, Xidian University, Xi’an, China; ^3^ Department of Electronic Engineering, City University of Hong Kong, Kowloon, Hong Kong SAR, China; ^4^ Center for Quantitative Biology, Peking University, Beijing, China; ^5^ Department of Biology, Southern University of Science and Technology, Shenzhen, China; ^6^ State Key Laboratory of Environmental and Biological Analysis, Hong Kong Baptist University, Kowloon, Hong Kong SAR, China

**Keywords:** FUCCI, cell cycle phase, embryogenesis, *C. elegans*, cell lineage

## Abstract

Early embryonic cell cycles usually alternate between S and M phases without any gap phase. When the gap phases are developmentally introduced in various cell types remains poorly defined especially during embryogenesis. To establish the cell-specific introduction of gap phases in embryo, we generate multiple fluorescence ubiquitin cell cycle indicators (FUCCI) in *C. elegans*. Time-lapse 3D imaging followed by lineal expression profiling reveals sharp and differential accumulation of the FUCCI reporters, allowing the systematic demarcation of cell cycle phases throughout embryogenesis. Accumulation of the reporters reliably identifies both G1 and G2 phases only in two embryonic cells with an extended cell cycle length, suggesting that the remaining cells divide either without a G1 phase, or with a brief G1 phase that is too short to be picked up by our reporters. In summary, we provide an initial picture of gap phase introduction in a metazoan embryo. The newly developed FUCCI reporters pave the way for further characterization of developmental control of cell cycle progression.

## Introduction

The developmental control of cell cycle progression is essential to ensure a balance between cell proliferation or growth and cell fate differentiation. Dysregulation of the balance may lead to catastrophes such as carcinogenesis or abnormal cell death. Unlike cell division in a single-celled organism or a cultured cell, which progresses through a full cell cycle with four phases: two gap phases, G1 and G2, that interrupt the DNA synthesis (S) phase from mitosis (M) phase, early embryonic division in most metazoans consists of M and S phases only without any gap phase (Farrell and O’Farrell, 2014). After certain rounds of synchronous divisions that are mostly driven by maternal factors, cell cycles become asynchronous, and gap phases are presumably introduced in a cell type- or developmental time-dependent manner. The transition from synchronous to asynchronous divisions coincides with zygotic genome activation when zygotic gene expression is initiated (Lee et al., 2014; Pálfy et al., 2017). However, the exact details of when the gaps are systematically introduced spatiotemporally during embryogenesis remains poorly defined.

One of the major difficulties in defining embryonic cell cycle phases is the lack of reliable reporters for a precise demarcation of cell cycle phases. The recent development of fluorescent ubiquitination-based cell cycle indicator (FUCCI) has paved the way for demarcation of cell cycle phases, especially in cultured cells (Sakaue-Sawano et al., 2008). The initial FUCCI system developed in human cell line relied on pairs of fluorescent proteins fused to the degrons derived from Cdt1 and Geminin proteins which are a DNA replication licensing factor and its inhibitor, respectively (Arias and Walter, 2007). The CDT1 level peaks in the G1 phase, and declines quickly after the initiation of the S phase due to the degradation by SCF^Skp2^ (Li et al., 2003; Nishitani et al., 2004); whereas the Geminin reaches a high level in the S and G2 phases, but falls to a low level in the late M and G1 phases due to the degradation by APC/C^Cdh1^ (McGarry and Kirschner, 1998; Arias and Walter, 2007). The reciprocal oscillations of the two factors during the cell cycle progression permit distinguishing cells in G1 phase from those in S/G2/M phase. Since their first introduction in human cell lines, FUCCI reporters have been developed in both cultured cells and intact animals (Sakaue-Sawano et al., 2013; Zielke et al., 2014; Bajar et al., 2016; Özpolat et al., 2017), greatly facilitating the study of regulation of developmental control over cell cycle progression. However, these reporters have not been generated in *C. elegans*, a well-established model organism.

Another difficulty in defining embryonic cell cycle phases lies in the systematic acquisition of the cellular accumulation patterns of fluorescence reporters with a high temporal resolution. This is because that cell divisions are rapid during early embryogenesis, which can be as fast as 8.6 min per generation (Foe and Alberts, 1983; Farrell and O’Farrell, 2014). Therefore, tracing the division of individual cells and measurement of reporter accumulation therein become a great challenge. *C. elegans* embryo is an excellent model for investigating gap phase introduction with cellular resolution. First, its embryo is transparent and develops with an invariant cell lineage within roughly 14 h at room temperature (Sulston et al., 1983), allowing live-cell imaging of the entire embryogenesis process at a high temporal resolution. Second, various automated tools have been developed to trace cell division and profile reporter accumulation with cellular resolution at 1.5-min intervals (Bao et al., 2006; Murray et al., 2008; Zhao et al., 2010a). Third, *C. elegans* embryogenesis demonstrates frequent division asymmetry in cell cycle length between two sister cells that develop into the same or different fate(s) (Sulston et al., 1983). We have previously shown that these asymmetries are primarily controlled by the regulatory factors determining fate differentiation (Ho et al., 2015). However, the method for systematic profiling of gap phase introduction has not been established in *C. elegans* though a postembryonic fluorescence reporters for cell cycle entry has been developed, which were based on reporter’s translocation rather than its accumulation or degradation to mark cell cycle commitment (vanRijnberk et al., 2017; Adikes et al., 2020). In addition, their dependence on the ratio between cytoplasmic and nuclear CDK abundance makes it not feasible for automated quantification of reporter intensity that relies on expression in nuclei, especially in late embryonic cells with minimal cytoplasm.

In this study, we determined the gap phase introduction for every cell during the embryogenesis of *C. elegans*. This was achieved by the development of multiple FUCCI reporters as a single-copy transgene in *C. elegans* (hereafter referred to as Worm-FUCCI), the degradation of which was biochemically and functionally validated. Aided by the automated tools for lineage and expression analysis (Bao et al., 2006; Murray et al., 2008), a combination of the individual FUCCI reporters with a lineaging marker allowed us to quantify the reporters’ lineal accumulation level for every cell at 1.5-min intervals throughout *C. elegans* embryogenesis, leading to a first-ever global picture of gap phase introduction throughout metazoan embryogenesis. We demonstrated that most embryonic cells appear to divide either with a very brief G1 phase or skipping the G1 and G2 phases altogether except one pair of cells, which apparently divide with a full cell cycle. We also demonstrated the potential of the reporters for cell cycle analysis during postembryonic development, including the development of germline and intestine. Availability of the Worm-FUCCI will aid future study of the coordination between cell division and fate differentiation during embryonic and postembryonic development.

## Materials and methods

### Worm strains and maintenance

All the animals were maintained on NGM plates seeded with OP50 at room temperature. The genotypes of the strains used in this paper were listed in the Supplementary Table S1. Imaging of postembryonic tissues was performed as described (Shao et al., 2013).

### DNA constructs

*his-72* promoter (2,349 bps from immediately upstream of its start codon), *pie-1* 3′UTR (787bp immediately after the stop codon), the nuclear localization signal (NLS) of EGL-13 (1-25aa), the degrons of CDT-1 (1-189aa) and CYB-1 (8-80aa) were amplified from the N2 genomic DNA, respectively. mCherry fragment was amplified from pCFJ104 (Frokjaer-Jensen et al., 2014), while eGFP was amplified from pZZ31 (Zhao et al., 2010a). The fusion cassette consisting of P*his-72*::mCherry::CDT-1(1-189aa)::*pie-1* 3′UTR was cloned into the *miniMos* vector pCFJ909 (Frokjaer-Jensen et al., 2014) to generate plasmid pZZ176 using Gibson Assembly according to the manufacturer’s description. Plasmid pZZ180 [P*his-72*::GFP::EGL-13 (1-25aa)::CYB-1 (8-80aa)::*pie-1* 3′UTR + unc-119(+)] was generated in the similar way as pZZY176. EGL-13 (1-25aa) was fused with the N-terminal of CYB-1 degron to serve as an NLS (Lyssenko et al., 2007). pZZ147 [P*his-72*::mCherry::EGL-13 (1-25aa)::CYB-1 (8-80aa)::*pie-1* 3′UTR + unc-119(+)] was made by cutting the pZZ141 (Supplementary Table S1) with ApaI and SpeI, respectively, to replace the HIS-24 coding region with the fusion between EGL-13 (1-25aa) and CYB-1 (8-80aa). The details of these constructs built in this study was listed in the Supplementary Table S2. Vector sequences and annotations can be found in the links below:

pZZ176—CDT-1D: https://benchling.com/s/seq-iUCSfeZIg DycQ5RkrD2s/edit pZZ147—CYB-1D: https://benchling.com/s/seq-mN00xlJVIAwAU66QJIDA/edit pZZ180—CYB-1DG: https://benchling.com/s/seq-LBAUfkXMHsEbc 8Z6uRE4/edit.

### 5-ethynyl-2′-deoxyuridine staining

Prior to EdU staining, L4 worms were fed with *perm-1* RNAi bacteria to permeabilize the eggshell as described (Carvalho et al., 2011). To confirm the permeabilization of eggshell, part of the embryos from the RNAi animals were stained with FM^®^ 4–64 dyes (Invitrogen). Embryos were retrieved from about 10 dissected worms and allowed to develop under the Boyd’s buffer/methyl cellulose for 3 h (Murray et al., 2006). Click-iT^®^ EdU Imaging Kit (Invitrogen) was used for EdU staining. After 3 h development, embryos were incubated with EdU for 15 min, followed by freeze-cracking, fixation and DAPI staining as described (Seydoux and Dunn, 1997). Embryos were then imaged for DAPI and mCherry accumulation of CDT-1^D^ and CYB-1^D^, followed by EdU staining with Alexa Fluor^®^ 647 using Leica SP5 Confocal microscope. Both DAPI and Alexa Fluor^®^ 647 in the same embryos were imaged again, using DAPI for cell alignment to overlay the Worm-FUCCI accumulation with EdU signal in the nuclei.

### RNA interference

RNAi against *cdt-2*, *fzr-1*, *cul-1*, *pat-3 or cyd-1* was performed by microinjection as described (Ho et al., 2015). RNAi against *ddb-1* was performed by feeding on the NGM plates supplemented with 50 μg/ml Ampicillin and 1 mM IPTG. RNAi against *perm-1* was performed similar to that of *ddb-1*, except the *perm-1* RNAi bacteria was diluted by “empty” vector (L4440) expressing bacteria in 1:6 ratio as described (Carvalho et al., 2011). The RNAi bacteria was derived from the Ahringer *C. elegans* RNAi feeding library (Kamath et al., 2001).

### Fluorescence microscopy for embryo

Micrographs of embryos were acquired with a Leica SP5 confocal microscope with an objective of ×63 magnification. Early embryos were dissected from young adult worms and mounted with Boyd’s buffer/methyl cellulose (Murray et al., 2006), and late embryos were picked from the NGM plate. For 3D imaging, GFP and mCherry were simultaneously illuminated with 488nm and 594 nm laser beams, respectively, and micrographs of their expression were collected with two separate hybrid detectors through a water immersion objective. Imaging setting was similar to what was used previously using a frame size of 712 × 512 pixels except the scanning speed was changed to 200 Hz (hz) (Ho et al., 2015). Laser compensation was applied during the stack acquisition to ensure the comparable brightness of the images acquired between the lower stack and upper stack. DIC images were acquired separately for a single focal plane typically in the middle of the embryo. For time lapse 3D imaging, it was performed as described (Murray et al., 2006) with the following modifications. Micrographs from 41 focal planes were collected consecutively for three embryos per imaging session from top to bottom of the embryo at an interval of about 1.5 min with a *Z*-axis resolution of 0.71 µm. Images were continuously collected for at least 200 time points. The entire imaging duration was divided into four blocks based on the time point, i.e., 1–60, 61–130, 131–200, and beyond 201. *Z* axis compensation was 0.4%–4% for 488 nm laser and 19%–95% for the 594 nm laser. The pinhole sizes for the four blocks were 2.3, 2.0, 1.6, 1.3 AU (airy unit). In general, the imaging duration for control embryos were around 6 h, whereas for the *cyd-1* RNAi embryos, the imaging duration was extended to 7 h to compensate the slower development of the embryo after RNAi. 3D projection was generated using Leica Application Suite X (LAS X).

### Imaging and data analysis beyond time point when embryo starts twitching

To image a developing embryo beyond the time point when it started twitching, knockdown of *pat-3* by RNAi was performed through microinjection. For automated lineaging of all embryonic cells up to 1.5-fold stage, the same settings were used as described above except the imaging duration was extended from 6 h to 9 h. For manual curation of the cells, V5QL/R, beyond the 1.5-fold stage, the RNAi embryo arrested at two-fold stage that specifically accumulated CYB-1^DG^ was traced backward till the time point when their exact identities were established through automated lineaging at approximately 1.5-fold stage.

### Automated lineaging and gene expression profiling

Strains expressing Worm-FUCCI were individually crossed either with strain RW10029 that broadly expresses a fusion between histone and GFP or RW10226 that broadly expresses a fusion between histone and mCherry, which were referred to as lineaging marker (Chen et al., 2018). FUCCI reporter contains mCherry or GFP was crossed with RW10029 and RW10226, respectively to allow automated cell tracing and lineal expression profiling. Both the lineaging marker and FUCCI reporter were rendered homozygous before automated lineaging and lineal gene expression profiling as described (Murray et al., 2008). Automated lineaging results were manually curated up to approximately 1.5-fold stage unless stated otherwise. All the expression data were normalized for the subsequent comparison.

### Transgenesis

The Worm-FUCCI strains carrying a single-copy transgene were generated using *miniMos* technique (Frokjaer-Jensen et al., 2014). Only strains with bright maternal and zygotic expression of Worm-FUCCI transgene were selected for the subsequent analysis (Supplementary Table S2). Transgene insertion site was mapped using inverse PCR as described (Frokjaer-Jensen et al., 2014). To facilitate simultaneous visualization of both degron reporters in the same animal, the transgenes consisting of P*his-72*::mCherry::CDT-1(1-189aa)::*pie-1* 3′UTR and P*his-72*::GFP::EGL-13(1-25aa)::CYB-1(8-80aa)::*pie-1* 3′UTR were rendered doubly homozygous by crossing.

### Fluorescence microscopy for postembryonic stages

Micrographs of larvae and gonads were acquired with tile scanning using the same confocal microscope as that for the embryo. Dissected gonads or intact adults were mounted with Boyd’s buffer/methyl cellulose (Murray et al., 2006) for imaging. Animals were mounted on a 1% agarose pad with 0.1 M sodium azide in M9 buffer for imaging with scanning speed of 200–400 hz depending on the size of the animals. For acquisition of 3D image stacks, imaging settings were similar to those used for the embryo except using 1 µm per z-step vs. 0.71 µm per z-step for the embryo.

### Time-lapse imaging of larvae

Synchronized L1 larvae were obtained through egg prep. Animal development time (in hour) was counted from the start of feeding. Five larvae were selected for imaging each hour before and after the feeding for a continuous duration of 13 h. Micrographs were acquired only for part of the intestine using 0.3 µm per z-step and 200 hz scanning speed. Micrographs of mCherry and DIC were collected simultaneously. A representative micrograph for the cells int2 and int3 were collected for illustration.

### Quantification and statistical analysis

To facilitate the comparison of accumulation intensities of the two reporters in the same cells from two different embryos throughout embryogenesis, two sets of reporter expression series ( E ) were acquired from an embryo expressing CDT-1^D^ $\left(E_{R};red;EmbryoR\right)$ and another embryo expressing CYB-1^D^ $\left(E_{G};green;EmbryoG\right)$ . The expression intensity, i.e., fluorescent signal intensity, of a cell ω at time point T was expressed by E(ω,T) , where T=1, 2, 3, … and its corresponding actual time was denoted by t (t=T·Resolution) . The confocal imaging started from a 4-cell stage embryo and ended in approximately 550-cell stage. A previously established quality control cell list, which provided a group of conserved and comparable developmental stages, was applied on both embryos (Cao et al., 2020; Guan et al., 2020). In brief, The quality control required the embryonic stage between $T_{4-cell}$ and $T_{∼350-cell}$ that must be imaged continuously, whereas the last co-existence time point of “ABa”, “ABp”, “EMS,” and “P2” cells were labelled as $T_{4-cell}$ and the first co-existence time points of “AB256”, “MS32”, “E16”, “C16”, “D8”, “Z2,” and “Z3” cells were labelled as $T_{∼350-cell}$ .

There were four types of unavoidable experimental variations that needed to be normalized before the comparison of reporter expressions.

A. Due to high dynamic range of the lineaging marker expression values, the entire imaging process was separated by multiple blocks that applying different pinholes. However, the changes of pinhole would also sharply and constantly change the absolute recorded value of FUCCI reporter expressions. To maintain the continuity and comparability of both sets of the reporter expression series, linear scaling on the expression data was subsequently applied on each change of pinhole at the exact time point $\left(T_{i}=60,\;130,\;200\right)$ . The proportional scaling coefficient $K_{E,i}$ was obtained by fitting the global FUCCI reporter expressions before and after the adjustment into similar and smooth values, according to $E{\left(\omega ,T\right)}^{\prime }=E\left(\omega ,T\right)\cdot K_{E,i}\;\left(T>T_{i}\right)$ ; here, $K_{E,i}=\frac{\underset{\omega \in \Omega }{\sum }E\left(\omega ,T_{i}\right)}{\underset{\omega \in \Omega }{\sum }E\left(\omega ,T_{i}+1\right)}$ , where Ω was the cells present at both time points $T_{i}$ and $T_{i}+1$ .

B. The expression level of both FUCCI reporters varied globally among the embryos. Therefore, the data from them were linearly scaled to a closer order of magnitude for better visualization and comparison. The proportional scaling coefficient $K_{E}^{\prime }$ was obtained by fitting the maximum reporter expression detected before the time point $T_{∼350-cell}$ in those two embryos into the same value, according to $E_{G}{\left(\omega ,T\right)}^{\prime }=E_{G}\left(\omega ,T\right)\cdot K_{E}^{\prime }$ ; here, $K_{E}^{\prime }=\frac{\underset{\omega \in \Omega^{\prime },\;T\leq T_{350}}{\sum }E_{G}\left(\omega ,T\right)}{\underset{\omega \in \Omega^{\prime },\;T\leq T_{350}}{\sum }E_{R}\left(\omega ,T\right)}$ , where $\Omega^{\prime }$ were the cells existing before the time point $T_{∼350-cell}+1$ .

C. The global variation in developmental paces between embryos, which was revealed by the slightly changeable cell cycle length ( C ), were frequently observed among individual embryos owing to multiple factors, such as individual fitness and the variation of room temperature. To normalize these variations, the cell cycle length of all cells that had complete lifespan and divided before $T_{∼350-cell}$ in both embryos were compared, and the relative growth rate $K_{C}$ of Embryo *G* compared to Embryo *R*, was calculated according to a method described previously (Guan et al., 2019). Then, the cell cycle length of all cells in Embryo *G* was transformed into $C_{G}{\left(\omega \right)}^{\prime }=C_{G}\left(\omega \right)/K_{C}$ .

D. Despite the global normalization on developmental pace, the cell cycle length of each specific cell would still be different in the two embryos. Hence, for each cell with complete lifespan recorded, its time points in Embryo *G* to fit the ones in Embryo *R* were linearly transformed by setting the actual time of appearance and the end of a cell that was totally same for both embryos, namely, $t_{G}{\left(\omega ,1\right)}^{\prime }=t_{R}\left(\omega ,T_{\min}\right),\;t_{G}{\left(\omega ,T_{\max}\right)}^{\prime }=t_{R}\left(\omega ,T_{\max}\right)$ . The transformation of each time point followed the formula $t_{G}{\left(\omega ,T\right)}^{\prime }=\left[t_{G}\left(\omega ,T\right)-t_{G}\left(\omega ,T_{\min}\right)\right]\cdot \frac{t_{R}\left(\omega ,T_{\max}\right)-t_{R}\left(\omega ,T_{\min}\right)}{t_{G}\left(\omega ,T_{\max}\right)-t_{G}\left(\omega ,T_{\min}\right)}+t_{R}\left(\omega ,1\right)\;\left(T_{\min}\leq T\leq T_{\max}\right)$ . For the cell without complete life span, its actual time of appearance was directly translated to Embryo *R*, and no linear scaling was performed.

All four experimental variations were normalized, and the expression data of Embryo *G* were aligned onto that of Embryo *R*, which served as a reference regarding both reporter expression level and developmental pace. Finally, the expression values of two reporters from two different embryos were drawn on a single linage tree or plotted individually for each cell.

## Results

### Generation of Worm-fluorescence ubiquitin cell cycle indicators

**FIGURE 1:**
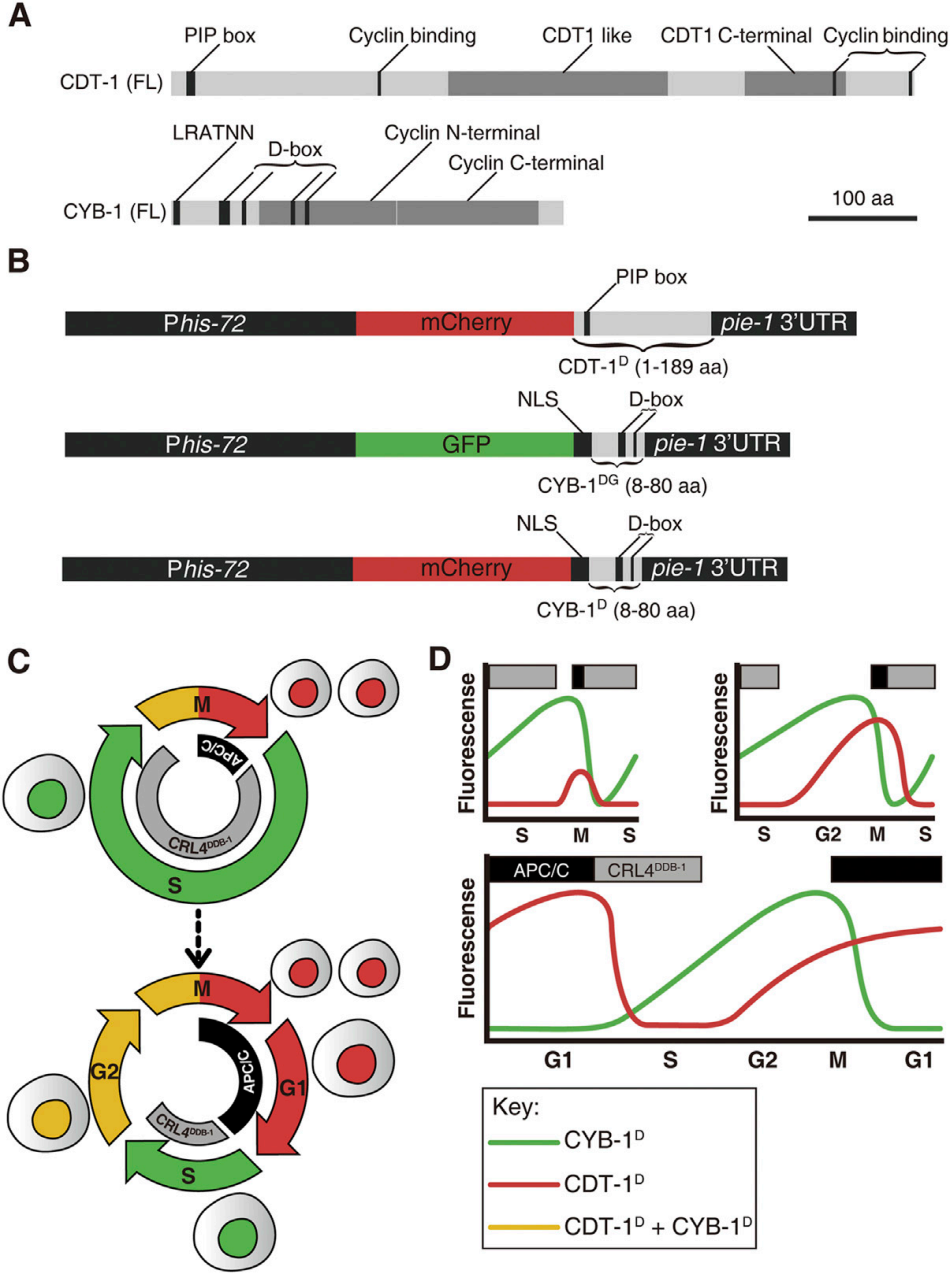
An overview of Worm-FUCCI design and expected marker expression dynamics in embryos. **(A)** Schematic representations of predicted domains in the full-length (FL) CDT-1 (top) and CYB-1 (bottom) proteins. Protein size in amino acid (aa) is shown in scale. PIP box: PCNA-interacting protein (CDT-1 degron). D-box: Destruction box (CYB-1 degron). LRVTRN: amino acid sequence of a chromosome localization motif. **(B)** Worm-FUCCI construct design. To ensure broad expression both maternally and zygotically, all expression cassettes are driven by a *his-72* promoter and flanked by a *pie-1* 3′ UTR. Top: the CDT-1 degron consisting of 1–189 aa of CDT-1 is fused to the C-terminus of mCherry (referred to as CDT-1^D^) to label the G0/G1 and G2 phases. The PIP box with the sequence of QTAVTDFF in the degron is targeted for degradation by CRL4^DDB−1^ complex. Middle: the CYB-1 degrons consisting of 8–80 aa of CYB-1 is fused to the C-terminus of GFP (referred to as CYB-1^DG^) to label the S and G2 phases. The putative D-boxes are targeted for degradation by APC/C^FZR−1^ complex. An NLS from EGL-13 (1–25 aa) is introduced between the GFP and the truncated CYB-1 to ensure nuclear localization of expression. Bottom: Same as the CYB-1 fusion with GFP except the substitution of the GFP with mCherry (referred to as CYB-1^D^). All of the fusion constructs are integrated into *C. elegans* genome as a single copy transgene *via miniMos* technique. **(C)** Current view of early embryonic cell cycle (top) and full cell cycle (bottom) with the reported degradation complexes for the degrons mentioned in “**(B)**” in *C. elegans* indicated, i.e., degradation of CDT-1 (red) and CYB-1 (green) by CRL4^DDB−1^ and APC/C complex at S and M/G1 phase respectively. **(D)** Predicted expression dynamics of the degron reporters mentioned in “B” during *C. elegans* embryogenesis. The degron reporters and their corresponding degradation complexes are color coded as in “**(C)**”. Three different cell cycle scenarios, i.e., with no gap phase, with only G2 phase, and with both G1 and G2 phases, are shown. Note that the introduction of G2 phase results in an earlier increase in CDT-1^D^ accumulation before M phase, whereas the introduction of G1 phase results in accumulation of CDT-1^D^ but degradation of CYB-1^D^ or CYB-1^DG^ after M phase. The upper rectangle boxes denote the timing of the active degradation complexes (gray: CRL4^DDB−1^; black: APC/C).

**FIGURE 2:**
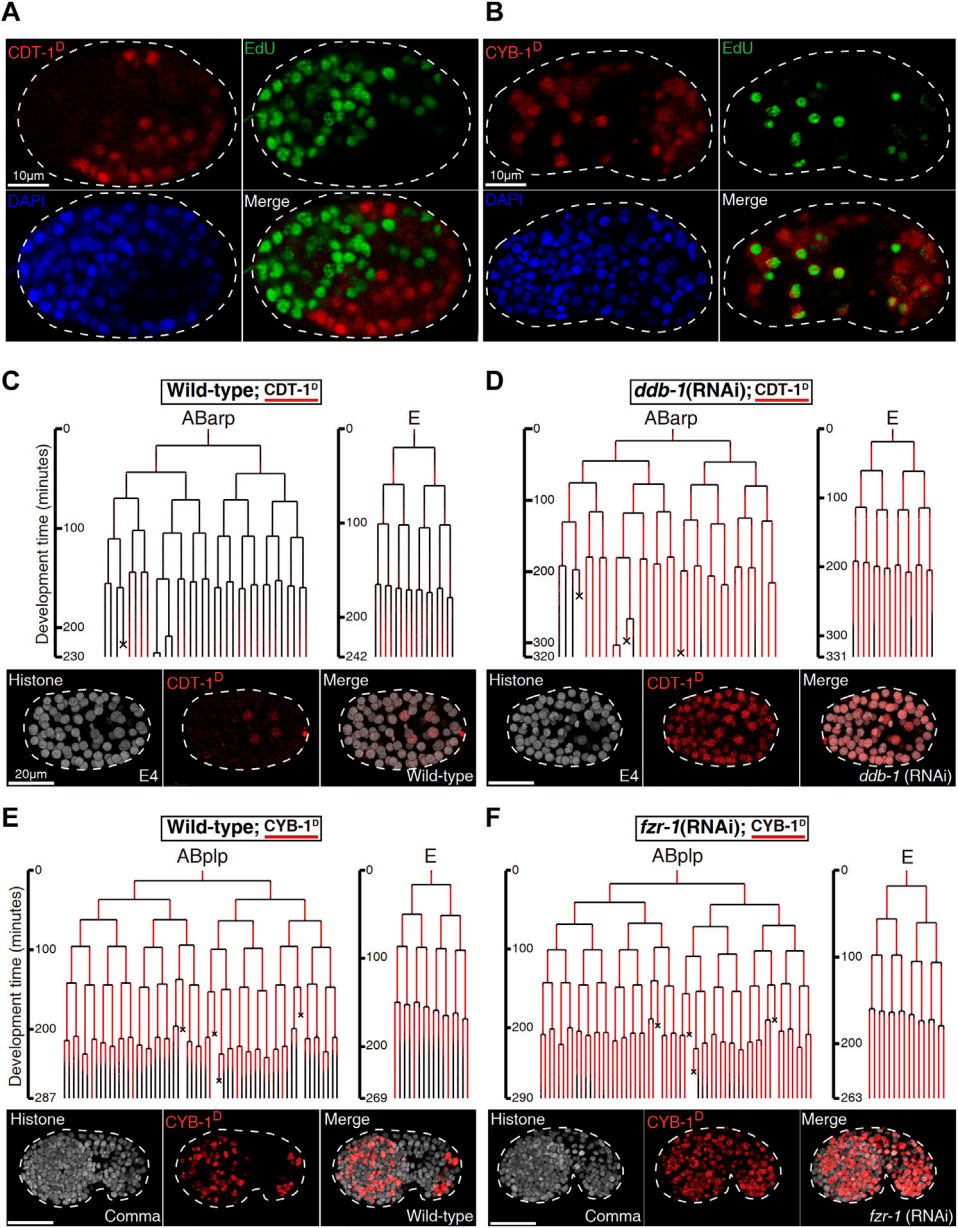
Biochemical and functional validation of Worm-FUCCI degradation (See also Supplementary Figures S3,S4). **(A)**. EdU staining of the embryo expressing mCherry::CDT-1_PIP-box (referred to as CDT-1^D^). Note that the localization of CDT-1^D^ (red) is mutually exclusive (merge) with the nuclei showing the staining of EdU (green) in a *perm-1* RNAi embryo. Nuclei stained with DAPI are shown in blue. **(B)**. Same as “**(A)**” except the embryo is expressing mCherry::CYB-1_D-box (referred to as CYB-1^D^). Note that the reporter-expressing cells (red) cover all the cells stained with EdU. The CYB-1-expressing cells that do not incorporated with EdU are presumably at G2/M phase. **(C)** Lineal expression (redness) of CDT-1^D^ in the sublineages, “ABarp” and “E” in a wild-type embryo. Development time starting from the birth of the ancestral cell of interest is shown on the left and cell deaths are indicated with an “×”. Epifluorescence micrographs for a representative time point at the “E4” stage [“E” divides into four daughters] are shown at the bottom. Histone, histone::GFP used for cell tracking during lineage analysis. **(D)** Same as “**(C)**” except the embryo is treated by RNAi against *ddb-1*. Note that expression onset of CDT-1 becomes much earlier and expression intensity becomes much higher in most cells after the RNAi. **(E)** Lineal expression of CYB-1^D^ in the sublineages of “ABplp” and “E” in a wild-type embryo. Development time and cell deaths are shown as in “**(C)**”. Epifluorescence micrographs for a representative embryo at comma stage are shown at the bottom. **(F)**. Same as “**(E)**” except that the embryo is treated by RNAi against *fzr-1*. Note that the degradation of the reporter during late embryogenesis is mostly abolished after the RNAi.

**FIGURE 3:**
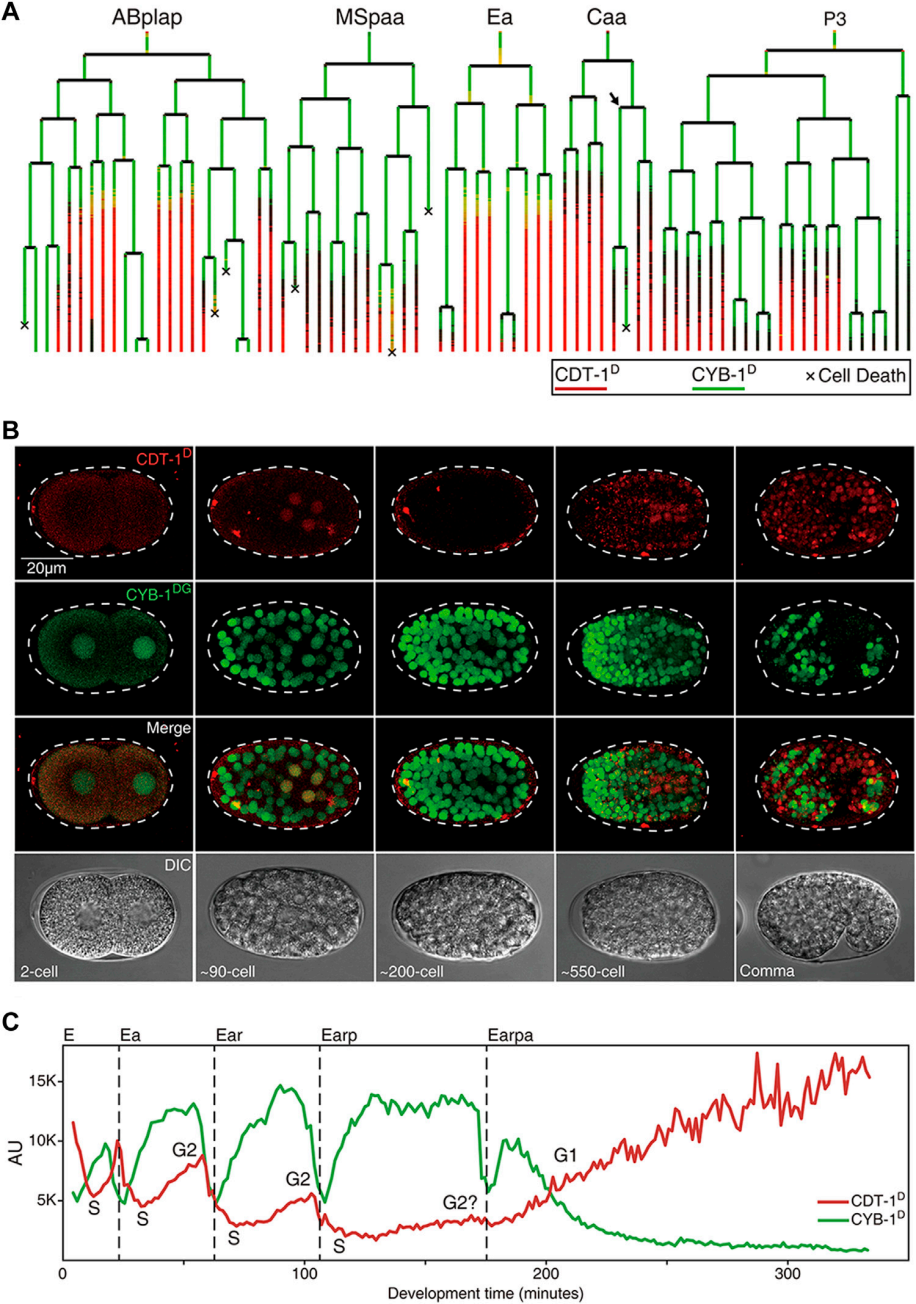
Expression dynamics of Worm-FUCCI in representative cell lineages during *C. elegans* embryogenesis (see also Supplementary Figures S1,S2). **(A)** Cell lineage trees showing the superimposed lineal accumulation of CDT-1^D^ (colored in red) and CYB-1^D^ (colored in green) in the representative sublineages of “ABplap”, “MSpaa”, “Ea”, “Caa”, and “P3”. Asynchrony of division between sister cells, “Caapa” and “Caapp”, is indicated with an arrow. Cell death is indicated with a “×“. Note the overall complementary expression patterns between the two reporters, i.e., the CYB-1^D^ is ubiquitously expressed during early embryogenesis, while the CDT-1^D^ is usually not expressed until a cell completes its last round of division. Long lasting CDT-1 expression is predictive of a cell cycle exit. **(B)** Epifluorescence micrographs of the embryos at different stages as indicated, which simultaneously express CDT-1^D^ and CYB-1^DG^ in the same embryo in some cells. The Nomarski micrographs of the same embryos are shown at the bottom. **(C)** Quantification of accumulation dynamics of CDT-1^D^ (red) and CYB-1^D^ (green) during the development of E lineage (only “Earpa” sublineage is shown). Normalized fluorescence intensity in the arbitrary unit (AU) is plotted on the *Y* axis. Development time from “E” to “Earpa” cells starting from the birth of “E” is plotted on the *X* axis. Cell cycle phases are indicated based on the expression dynamics of the two reporters. Division time point of each cell are indicated with a dashed line.

To map the developmental introduction of gap phases during *C. elegans* development, we built a worm version of FUCCI, which consisted of degron reporters derived from two proteins: *C. elegans* orthologues of human CDT1 and cyclin B1 (CCNB1) protein, i.e., CDT-1 and CYB-1. Initial CDT1 derived FUCCI relied on one of its two degrons, i.e., Cy motif (Sakaue-Sawano et al., 2008). Although the degron is absent in the *C. elegans* CDT-1, the other degron of CDT1, PIP box, is present. As the PIP degron is rapidly degraded by CUL4^Ddb1^ during the S phase onset in humans (Sakaue-Sawano et al., 2017), CDT1 is extremely abundant in the G1 phase but barely detectable in the S phase. This degradation pathway was also shown to be conserved in *C. elegans* (Zhong et al., 2003; Kim and Kipreos, 2007; Özpolat et al., 2017). A recent study demonstrated the superiority of the PIP-containing degron as a G1-specific degron over the Cy motif, as it provided a sharper boundary between G1 and S phases in a human cell line (Sakaue-Sawano et al., 2017). To generate a G1-and G2-specific reporter in *C. elegans*, a sequence consisting of 1–189 CDT-1 amino acids that carried the PIP box but lacked the putative cyclin binding motif was fused with the C-terminus of mCherry (referred to as CDT-1^D^ hereafter) (Figures 1A,B). To achieve broad spatial and temporal expression, the fusion was flanked by a *his-72* promoter and a *pie-1* 3′ untranslated region (UTR). The *his-72* promoter drives strong zygotic expression but is less capable in driving germline expression (Ooi et al., 2006). The inclusion of a *pie-1* 3’ UTR has been shown to significantly boost germline expression (Merritt et al., 2008; Zhao et al., 2010a) (Figure 1B). Therefore, the reporters are expected to show strong and broad expression in both germline and Soma in the absence of robust degradation. The truncated CDT-1 sequence seems to contain a cryptic NLS that directs the reporters into nuclei (Figures 2A, 3B). The cassette was introduced into random locations of the *C. elegans* genome as a single copy using the *miniMos* technique (Frokjaer-Jensen et al., 2014). The transgenic strain with the brightest and broadest expression but without apparent developmental defect was selected for the subsequent analysis. Nuclear expression is important to the subsequent analysis of reporter expression based on automated lineaging and expression profiling technologies (Bao et al., 2006; Murray et al., 2008). Broad accumulation of the reporter was observed for nearly all cells after they completed their last round of division during embryogenesis (Supplementary Figure S1). The accumulation dynamics of the reporter were expected to mimic those of their human equivalents, i.e., accumulation of the reporter was high in the G1 but completely absent in the S phase, followed by accumulation starting from the G2 and peaking again in the G1 phase (Figures 1C,D).

To develop a second reporter in assisting the CDT-1^D^ in defining cell phase boundaries, we attempted to use a *C. elegans* equivalent as that in humans, i.e., Geminin. However, the Geminin orthologue (*gmn-1*) was barely identifiable by sequence alignment in *C. elegans*, although its function appeared to be conserved (Yanagi et al., 2005). We therefore used a highly conserved degron of cyclin B1 (CYB-1), whose *Drosophila* orthologue has been demonstrated to show accumulation dynamics that is comparable to those of human Geminin (Zielke et al., 2014). The N-terminal sequences of both *C. elegans* CYB-1 and its mouse orthologue, cyclin B1, contain a degron called the destruction box (D-box) (Figure 1A). The mouse cyclin B1 was demonstrated to be degraded by APC/C^Fzr/Cdh1^ in human cell line (Zur and Brandeis, 2002). The CYB-1 N-terminal sequence (8–80 amino acid) contains the first two D-boxes but lacks the putative mitotic chromosome association motif (Pfaff and King, 2013). The full N-terminal sequence also carries another two putative D-box degrons located within the cyclin N-terminal domain. We omitted the two D-box degrons within the cyclin N-terminal domain to avoid the potential functional interference of the native protein, which may lead to abnormal degradation. The truncated fragment was fused with the C-terminus of either GFP (referred to as CYB-1^DG^) or mCherry (referred to as CYB-1^D^) (Figure 1B). The truncated CYB-1 sequence contains the first two D-boxes with the sequences of REILALKPSN and RINL, respectively. Again, the fusion was flanked by the same regulatory sequences as those for the cassette CDT-1^D^ to achieve broad expression in germline and Soma. However, the transgenic animals generated with these constructs were not expressed in the nuclei (Wang et al., 2013). An NLS sequence derived from EGL-13 (Lyssenko et al., 2007) was fused between the fluorescence protein sequence and the degron sequence, resulting in bright expression of the single-copy transgenes in the nuclei (Figures 2B, 3B). In contrast with the high abundance of CDT-1^D^ in the G1 phase, the accumulation of the CYB-1^D^ was expected to be barely detectable in the G1 phase, but to accumulate from the S phase and peak at the start of the G2 phase (Figures 1C,D). In summary, we created the transgenic strains that carry a single copy of reporters fused with degrons from CDT-1 or CYB-1, which we referred to as Worm-FUCCI. The high abundance of CDT-1^D^ accumulation in the absence of the CYB-1^D^ or CYB-1^DG^ would be indicative of the G1 or G0 phase of a cell; whereas the absence of the CDT-1^D^ with the initial accumulation of CYB-1^D^ or CYB-1^DG^ would be indicative of the S phase; and a high abundance of both degron reporters would be indicative of the G2 phase (Figures 1C,D).

### Validation of degradation dynamics of the Worm-fluorescence ubiquitin cell cycle indicators

To validate whether the Worm-FUCCI was temporally degraded as expected, we first examined whether the CDT-1^D^ was absent but the CYB-1^D^ was present in the S phase of embryonic cells. To this end, we investigated the concurrence between the incorporation of 5-ethynyl-2′-deoxyuridine (EdU) and the accumulation of the CDT-1^D^. EdU was expected to be incorporated into DNA only at the S phase. To permeabilize the egg shell for EdU, we partially inactivated *perm-1* by RNA interference (RNAi) as reported previously (Carvalho et al., 2011). As expected, the accumulation of CDT-1^D^ was mutually exclusive to the staining of EdU in nearly all embryonic cells (Figure 2A; Supplemenary Figure S4A), while the accumulation of CYB-1^D^ was mostly overlapped with the staining of EdU (Figure 2B; Supplemenary Figure S4B). Because the CYB-1^D^ was expected to be accumulated also in the G2 phase, those cells showing CYB-1^D^ accumulation but no staining of EdU were expected to be at the G2 phase.

We next functionally validated the S phase-specific degradation of CDT-1^D^, which was reported to be degraded by cullin 4-based complex, i.e., cullin-RING ligase (CRL) coupled with adaptor protein DDB-1 (together referred to as CRL4^DDB−1^), in *C. elegans* (Kim and Kipreos, 2007). We first performed RNAi against the gene encoding the adaptor of CUL-4, *ddb-1*, using a strain that simultaneously expressed the degron reporter and a lineaging marker, i.e., histone::GFP (Murray et al., 2008). We then performed automated lineaging and lineal gene expression analysis using this strain with and without RNAi treatment as described (Zhao et al., 2010b). We did not detect any accumulation of CDT-1^D^ in most cells until about 550-cell stage of embryogenesis in a control embryo, which refers to a wild-type embryo without any perturbation except transgenesis-related genetic modification (Figures 2C; Supplemenary Figure S1). In contrast, the RNAi led to the continuous accumulation of the degron reporter much earlier than that in the control embryo (Figure 2D), confirming that the degron had been targeted by the degradation pathway involving DDB-1 as expected. In addition to the adaptor DDB-1, CDT-2 was shown to function as a substrate recognition subunit in the CRL4 ubiquitin ligase for CDT-1^D^ degradation (Kim et al., 2008). RNAi against *cdt-2* led to an increased accumulation of CDT-1^D^ in most cells when compared with the control embryo (Supplemenary Figure S3), further confirming that the CDT-1^D^ was degraded by the cullin-RING ubiquitin ligase, CRL4^DDB−1^, in *C. elegans*.

The CYB-1^D^ was expected to accumulate in any cells with an active S and/or G2 phase and to abolish its accumulation in embryonic cells starting from M phase until the end of G0/G1 phase. The transgenic strain carrying CYB-1^D^ construct indeed demonstrated CYB-1^D^ accumulation throughout early embryonic cell cycle but degraded in most of the late embryonic cells (Figure 2E; Supplemenary Figure S2). Notably, CYB-1^D^ also demonstrated an unexpected accumulation during and after the M phase. This could be due to the omission of other degrons within the CYB-1^D^ sequence, which led to a deviation from its native degradation pattern. A previous study showed that the mammalian CYB-1 orthologue, cyclin B1 was first targeted by APC/C^Fzy/Cdc20^ for degradation from prometaphase up to late M phase or anaphase (Clijsters et al., 2013). The degradation was relayed by APC/C^Fzr/Cdh1^, which remained active till late G1 phase (Zur and Brandeis, 2002). Since the functions of both degradation complexes were known to be conserved in *C. elegans* (Fay et al., 2002; The et al., 2015; Kipreos and van denHeuvel, 2019), we reasoned that one the one hand the improper perdurance of CYB-1^D^ through M phase till the early stage of next cycle (Figure 2E), could be due to the failure of the Cdc20/Fzy orthologue (FZY-1) to target the truncated cyclin for degradation because of missing degrons. On the other hand, it was possible that the Cdh1/Fzr orthologue (FZR-1) may not be robust enough to completely degrade CYB-1^D^ before the end of the M phase. In addition, mutation in *fzy-1* led to early embryonic arrest (Tarailo et al., 2007), making it infeasible to perform lineage analysis. We therefore performed RNAi against *C. elegans fzr-1* followed by automated lineage and lineal gene expression analysis to see if CYB-1^D^ was indeed targeted by APC/C^FZR−1^ for degradation. Instead of the absence of accumulation in the wild-type embryo around 550-cell stage, we observed a substantial increase in the accumulation of CYB-1^D^ in most of the embryonic cells that completed their last round of division (Figure 2F), confirming that the *C. elegans* D-boxes within CYB-1^D^ was targeted by the degradation complex APC/C^FZR−1^.

### Worm-fluorescence ubiquitin cell cycle indicators detects a G1 phase present at only a few embryonic cells before they complete their last round of division

Equipped with the Worm-FUCCI reporters that showed expected accumulation dynamics, which were predictive of cell cycle phases, we set out to systematically determine the gap phase introduction during *C. elegans* development, with a focus on embryonic cell cycles. Aided by the automated lineaging and gene expression profiling technologies (Bao et al., 2006; Murray et al., 2008), we took time-lapse 3D images to trace cell lineage and acquire lineal expression up to the 1.5-fold stage of embryogenesis, upon which lineaging analysis became impractical due to twitching (Supplementary Figures S1,S2, Supplementary Movie S1). Notably, most of the embryonic cells had completed their last rounds of division by this stage with a few exceptions discussed below. To facilitate the comparison and quantification of accumulation of CDT-1^D^ and CYB-1^D^, we superimposed the lineal accumulation patterns of the two reporters in a single lineage tree (Figure 3A) as described previously (Murray et al., 2008). To allow visualization of the two reporters in the same embryo, we crossed the transgenic CDT-1^D^ and CYB-1^DG^ alleles into the same animal in which both were rendered homozygous (Figure 3B).

Strikingly, the accumulation patterns of the two reporters barely overlapped but were mostly complementary both spatially and temporally in nearly all embryonic cells, i.e., CYB-1^D^ accumulated broadly during early embryogenesis, whereas CDT-1^D^ did not accumulate in most of the embryonic cells until they had completed their last round of embryonic division (Figures 3A,C). These results suggest that most embryonic cell cycles progress mostly with the S and M phases only and arrest in the G0 or G1 phase after they have completed their last rounds of division. However, it remains possible that there might be some brief G1 phases present in the embryonic cells, but may not be picked up by our reporters due to the limited temporal resolution imposed by the mCherry maturation time and the degradation efficiency of the reporters. Consequently, the CDT-1^D^ expression signal enables the accurate prediction of cell cycle exit, i.e., the CDT-1^D^ signal is not observed until a cell completes its last round of division, after which the CDT-1^D^ signals are seen in nearly all embryonic cells in a cell fate-independent way (Figures 3A,B; Supplementary Figure S1). As our reporters could not distinguish the G1 phase from the G0 phase, we assume that most CDT-1^D^-expressing cells that have completed the last round of division in their life cycle are arrested in the G0 phase.

To functionally validate this observation, we used RNAi to inactivate *cul-1*, which encodes a key cell cycle regulator required for cell cycle exit. Its perturbation was expected to prevent the embryonic cells from entering G0 or G1 phase (Kipreos et al., 1996). As expected, only CYB-1^D^ accumulation was detected in the perturbed embryos even they died (Supplementary Figure S5), indicating that cells of the RNAi embryo failed to arrest in the G0 or G1 phase. Due to the inability of our markers in distinguishing G0 from G1 phase, further resolving the arresting phase in the dead embryo requires independent markers, such as those that have been recently developed (Adikes et al., 2020).

We observed an inconsistence of Worm-FUCCI in demarcating cell cycle progression of germline progenitor P4 and its two daughter cells, “Z2.” and “Z3.” Despite embryonic arrest of the “Z2” and the “Z3,” CDT-1^D^ accumulation was not observed, while CYB-1^D^ accumulation was lost after the 350-cell stage (Figure 3A, Supplementary Figures S1,S2). This appeared to be due to the inability of the *his-72* promoter to drive the zygotic expression of Worm-FUCCI in the germline progenitors of the embryo rather than its cell cycle-specific degradation (Murray et al., 2006; Ooi et al., 2006; Zhao et al., 2010a). Therefore, a promoter that is able to simultaneously drive reporter expression in the germline progenitors is necessary to indicate cell cycle progression therein.

### Division asynchronies between sister cells seem mainly due to the differential durations of the S phase during embryogenesis

Unlike synchronous cell divisions in the early embryo of many other metazoans, *C. elegans* embryonic cell division is asynchronous from the very first division and becomes more obvious during late embryogenesis (Bao et al., 2008; Budirahardja and Gonczy, 2008; Rivers et al., 2008). As only CYB-1^D^ accumulation was seen in the sister cells between which division asymmetry in cell cycle length was observed (Figure 3A, Supplementary Figures S1,S2), lack of accumulation of CDT-1^D^ indicating that no involvement of G1 phase in the asymmetry. For example, the division asymmetry between the sister cells “Caapa” and “Caapp” is around 50 min during which accumulation of CDT-1^D^ or degradation of CYB-1^D^ is expected to be observed, but we did not observe either of them. Then the asymmetry could be due to differential duration of M, S or G2 phase. However, the entire duration from chromatin condensation to mitosis (assumed to be the M phase) was only around two minutes (Suppmentary Figure S6), suggesting that the differential duration of the M phase between the two sister cells was unlikely to be responsible for the observed asymmetry. Therefore, a differential duration of S or G2 phase was responsible for the observed division asymmetries in cell cycle length. However, we expected accumulation of both CDT-1^D^ and CYB-1^D^ during the G2 phase. Presence of CYB-1^D^ accumulation only suggests that it was the differential duration of the S phase that was responsible for most of the observed division asymmetries in cell cycle length.

### G2 phase is first introduced in the intestine precursors during embryogenesis

The “E2” (two daughters of “E,” the intestine progenitor) and “E4” (four granddaughters of “E”) of the intestine primordium (“E”) were found to accumulate both CDT-1^D^ and CYB-1^D^ simultaneously at the late stage in their cell cycles, indicated that a G2 phase was introduced in these cells (Figures 3A,C). Consistent with this, a previous study with DAPI staining also demonstrated that “E2” cells acquired the G2 phase (Edgar and McGhee, 1988). However, the accumulation of CDT-1^D^ was become barely detectable in E8 (eight daughters of E4), which suggests that the G2 phase was lost (Figures 3A,C). It was also possible that the accumulation level of CDT-1^D^ in the E8 cells was too low to be detected. Alternatively, maternal contribution of either mRNAs or proteins or both could boost the abundance of CDT-1^D^. Consistent with this, *ddb-1* RNAi led to robust accumulation of CDT-1^D^ in E8 cells when it lacks degradation (Figure 2D). It remains possible that the degradation of CDT-1^D^ was so robust that the accumulation window of CDT-1^D^ was too short to be visualized due to relatively long mCherry’s maturation time, whereas the G2 duration in the E2 or E4 cells could be substantially longer than that in the E8 cells. Intriguingly, upon the E16 (daughters of E8) stage, most of the cells arrested in the G1 phase unless they underwent one more round of embryonic division, as judged by the accumulation of the two degron reporters (Figure 3A, Supplementary Figures S1,S2), which mirrors the cell cycle characteristics of most other embryonic cells.

### Worm-fluorescence ubiquitin cell cycle indicators reporters reliably detect an obvious G1 and G2 phase only in the last round division of “ABplapapaa” and “ABprapapaa” during embryogenesis

**FIGURE 4:**
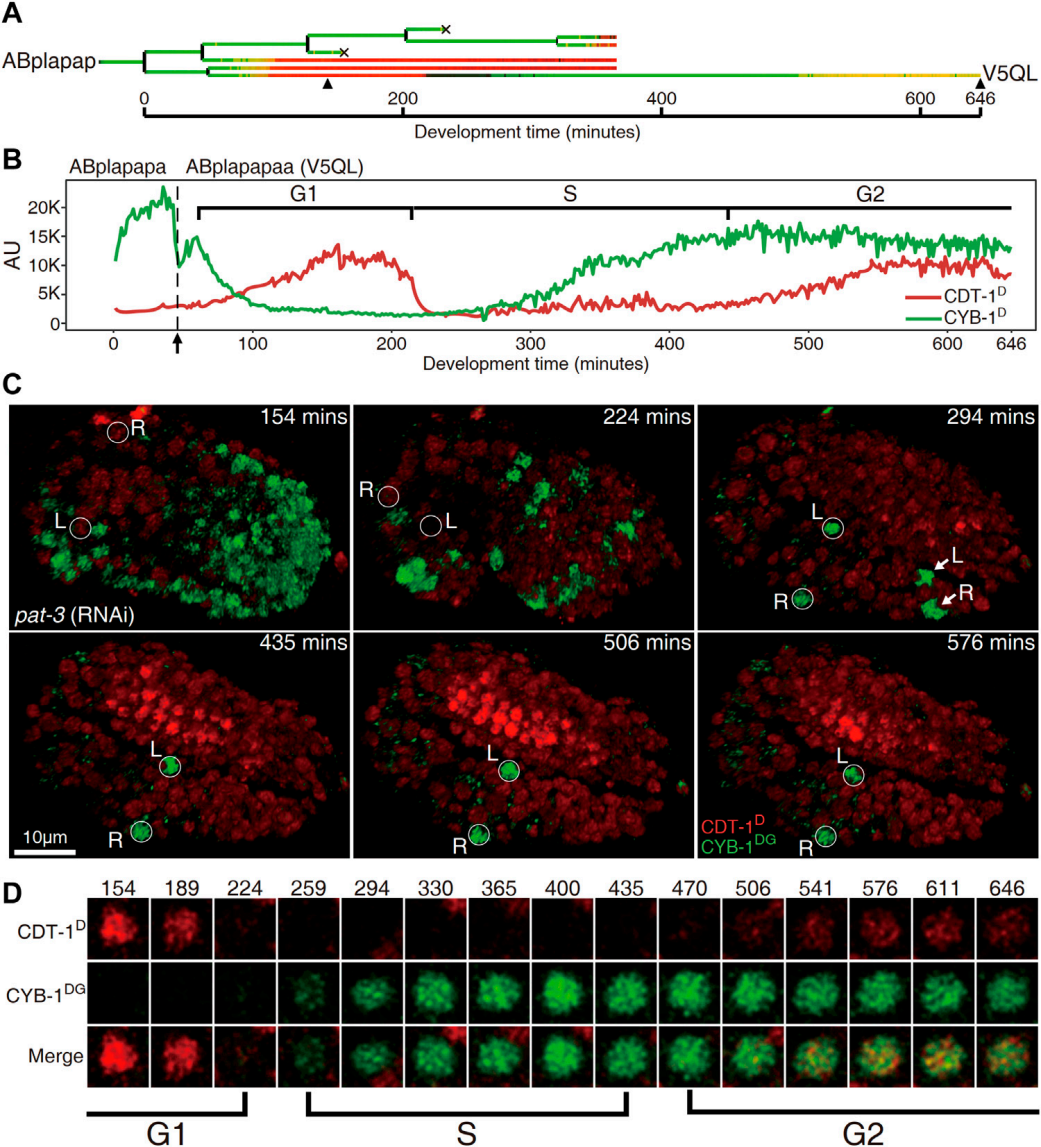
Cells “V5QL” (“ABplapapaa”) and “V5QR” (“ABprapapaa”) divide with a full cell cycle during *C. elegans* embryogenesis. **(A)**. Lineal accumulation of CDT-1^D^ (red) and CYB-1^D^ (green) in the sublineage of “ABplapap,” the grandparent of V5QL in the embryos treated with RNAi against *pat-3* to inhibit twitching. All cells are curated up to 365 min after the birth of “ABplapap” or up to their apoptotic cell death except the “V5QL” cell, which is curated up to 646 min after its birth (see Materials and Methods). For simplicity, only the expression of “V5QL” is shown. **(B)**. Quantification of accumulation of CDT-1^D^ (red) and CYB-1^D^ (green) in the “V5QL” cell and its parent (“ABplapapa”). Fluorescence intensity in the arbitrary unit (AU) is plotted on the *Y* axis and development time from the birth of “ABplapap” on the *X* axis. Durations of cell cycle phases are indicated with scaled bar above based on the expression dynamics of the two reporters. Division time point of “ABplapapa” is indicated with an arrow. **(C)** Expression dynamics of CDT-1^D^ and CYB-1^DG^ in a late embryo treated with RNAi against *pat-3* (see also Supplementary Movies S1,S2). Shown is the 3D projection of epifluorescence micrographs at six time points as indicated with CYB-1^DG^ (green) and CDT-1^D^ (red). The embryo is oriented so that both “V5QL” and “V5QR” (highlighted with white circles and indicated with L and R, respectively) are located at the posterior of the embryo. Development time is shown as in “**(B)**”. Note that the CYB-1^DG^ is degraded in most cells at 1.5 fold stage (224 min) but is highly accumulated only in “V5QL(R)” and “ABpl(r)apappppa” (indicated with L and R, respectively) upon two-fold stage (294 min). The CYB-1^DG^ becomes completely degraded in the latter two cells about 140 min later. Also note that the CDT-1^D^ is accumulated at the bean stage (154 min) and becomes completely degraded upon 294 min in the “V5QL(R)” cells. Most cells accumulate a high level of CDT-1^D^ during late embryogenesis, indicating their arrest at G1 or G0 phase. **(D)** Magnified views of the “V5QL” cells shown in “C” that simultaneously accumulate CDT-1^D^ (red) and CYB-1^DG^ (green) over development. Development time in minute is shown on the top. The first and the last time points correspond to those indicated with black triangles in “**(A)**”. Cell cycle phases are indicated based on reporter expression dynamics.

As stated above, most of embryonic cells did not accumulate CDT-1^D^ until they had completed their last round of embryonic division. Notably, a few cells did not complete their last round of division until about one hour before hatching, meaning that they had a very long cell cycle duration (Supplementary Figure S7) (Sulston et al., 1983). We wondered whether any gap phases had been introduced in these cells. However, these cells continued to divide after the embryo had started twitching, presenting a significant challenge to the live-cell imaging required for the subsequent lineaging analysis (Supplementary Movie S1) (Murray et al., 2008). To facilitate imaging during development of these cells, we depleted the activity of *pat-3*, which encodes a *β*-integrin subunit required for normal muscle filament assembly and function (Gettner et al., 1995). This depletion permitted the perturbed embryo to continue developing without twitching and rotating until its arrest at around the 2-fold stage (Supplementary Movie S2). This allowed us to trace cell division beyond the 1.5-fold stage using extended imaging time. As expected, we observed a clear two-way accumulation dynamic of CDT-1^D^ in one cell pair, consisting of “ABplapapaa” and “ABprapapaa,” referred to as “V5QL” and “V5QR,” respectively hereafter, which divided about one hour before hatching (Supplememtary Figure S7). CDT-1^D^ accumulated soon after birth of the two cells, peaked and got completely degraded at around 150 and 220 min after their birth, respectively, in both cells. Importantly, the CDT-1^D^ accumulated again at roughly 450 min and peaked around 550 min after their birth till the end of the imaging process (Figures 4A,B). The two cells developed into symmetrical cell fates, i.e., dividing into “V5L” and “QL” cells (postembryonic blast cells for hypodermis and neuron, respectively) or “V5R” and “QR” cells, respectively, during embryogenesis. The two cells resume cell division during postembryonic development, giving rise to hypodermal or neuronal cells (Sulston et al., 1983). Notably, “V5QL” and “V5QR” were the last cells to divide during embryogenesis (Supplememtary Figure S7, Supplementary Movie S1) (Sulston et al., 1983). Immediately after the disappearance of CDT-1^D^, CYB-1^D^ started to accumulate, and peaked about 7 h after their birth in the same cells (Figures 4A,B). The presence of CDT-1^D^ but the absence of CYB-1^D^ in this long duration (roughly two hours) indicated a G1 phase, whereas the absence of CDT-1^D^ indicated an S phase here. The simultaneous accumulation of the two degron reporters indicated a G2 phase.

In addition to “V5QL” and “V5QR”, coelomocyte precursors, “MSapapaa” and “MSppapaa,” also completed their last round of embryonic division relatively late during embryogenesis, which has long been speculated to divide with G1 phase (Boxem and van denHeuvel, 2001; Yanowitz and Fire, 2005; Kipreos and van denHeuvel, 2019). However, only CYB-1^D^ but not CDT-1^D^ showed accumulation in these two cells before their division (Supplementary Figures S8A,B), suggesting that an elongated S phase was responsible for their relative long cell cycle duration. It is also possible that the accumulation window of CDT-1^D^ may be too short to be detected by our reporters. To test this, we performed RNAi against *cyd-1*, which is expected to lead to arrest of these cells at the G1 phase before their last round of cell division during embryogenesis. As expected, despite the lack of CDT-1^D^ accumulation in the control embryo, we detected an obvious accumulation of CDT-1^D^ and degradation of CYB-1^D^ in one of the coelomocyte precursors after the RNAi (Supplementary Figures S8C,D), which strongly argue the presence of a brief G1 phase in these cells that is beyond of reach by the reporters in the control embryo.

Most other cells arrested at the G0 or G1 phase up to the late “2-fold” stage (actually beyond the 2-fold stage in a control embryo due to embryonic arrest caused by the depletion of *pat-3*), which can be judged by the apparent accumulation of CDT-1^D^. “V5QL” and “V5QR” were the only two cells still expressing CYB-1^DG^ in the late “2-fold” stage (Figures 4C,D, Supplementary Movie S2). Based on the degradation characteristics of the two degron reporters, our Worm-FUCCI demonstrated obvious G1 and G2 phases only in two embryonic cells, “ABplapapaa” and “ABprapapaa” cells, which apparently develop with a full cycle consisting of G1, S, G2, and M phases. Ability of our degron reporters to detect G1 in V5QL/R cells but not in coelomocyte precursors indicates that G1 phase duration is not uniform and can be quite short in cells that have to wait a long time before dividing.

### Accumulation dynamics of Worm-fluorescence ubiquitin cell cycle indicators during postembryonic development

The Worm-FUCCI showed accumulation dynamics not only during embryogenesis, but also during postembryonic development (Supplementary Figures S9A–D). For example, as expected, CDT-1^D^ but not CYB-1^DG^ was accumulated in nearly all the cells at dauer stage, indicating these cells arrested at G0 or G1 stage (Supplementary Figure S9E). Given that there is a wide spectrum of cells showing accumulation dynamics during postembryonic development, including vulva, seam cells and neurons, here we only explored on the accumulation dynamics in the germline and the intestine of larvae.

**FIGURE 5:**
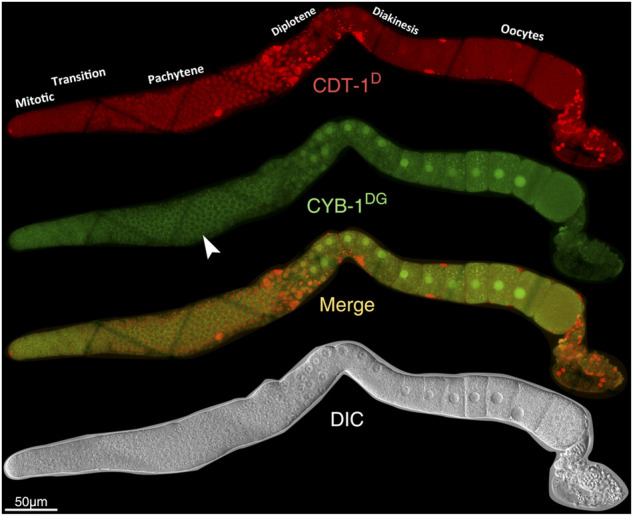
Overview of the accumulation dynamics of CDT-1^D^ (red) or CYB-1^DG^ (green) in adult germline. From top to bottom: Epifluorescence micrographs of CDT-1^D^, CYB-1^DG^, the merge between the two, and DIC micrograph. Specious accumulation of CYD-1^DG^ in the cytoplasm of pachytene stage (indicated with white arrowhead) is an artifact from autofluorescence rather than the accumulation of CYD-1^DG^ itself (see also Supplementary Figures S10,S11).

Both CDT-1^D^ and CYB-1^D^ or CYB-1^DG^ demonstrated accumulation dynamics in the germline (Figure 5; Supplementary Figures S10–12). Notably, their accumulation patterns were largely non-overlapping between each other. For example, the reporter CDT-1^D^ showed overall accumulation mostly at the pachytene stage; whereas the reporter CYB-1^D^ showed accumulation mostly at the diplotene and diakinesis stages during oogenesis (Figure 5). The specious non-nuclear accumulation of CYB-1^D^ in the mitotic zone and at the pachytene stage (Figure 5) apparently resulted from the autofluorescence of the tissue rather than from the CYB-1^D^ itself. This was because that the epifluorescence signals in these regions were comparable to those in the wild-type (N2) germline (Supplementary Figure S11). Despite the lack of CYB-1^DG^ accumulation in the mitotic zone of the germline, the reporter CYB-1^D^ did show additional nuclear accumulation in the mitotic zone and during oogenesis (Supplementary Figure S10B,S12). The accumulation in the mitotic zone became barely detectable upon the meiotic prophase entry that extended through late pachytene, and this accumulation drop was apparently not due to a shift to cytoplasmic enrichment (Supplementary Figure S12). Therefore, despite the consistent accumulation patterns in the Soma between CYB-1^D^ and CYB-1^DG^, the two reporters did show discordant accumulation patterns in the germline, making the CYB-1-based reporters unsuitable for deducing gap phase of cell cycle progression in the tissue.

Cell cycle progression in the mitotic zone occurs rapidly, continuously, with little or no time spent on the G1 phase (Fox et al., 2011). Consistent with this, few of the cells in this region showed an accumulation of CDT-1^D^ only, which was indicative of G1 phase. Most cells in the mitotic zone also showed an accumulation of CYB-1^D^ (Supplementary Figure S10). Only a very small portion of cells in the region did not show accumulation of CDT-1^D^, and most cells also show accumulation of CYB-1^D^ (Supplementary Figure S10). The results suggested that most of the cells were at G2 phase of cell cycle in the mitotic zone.

CDT-1^D^ accumulation was high in the dorsal and ventral intestine cells, including int2 (referred to as “int2D” and “int2V” hereafter, respectively) and int3 (referred to as “int3D” and “int3V” hereafter, respectively), of the synchronized L1 animals 5 h after feeding (Supplementary Figure S13). The “int2D/V” cells are known to undergo DNA endoreplication while the int3D/V cells to undergo both DNA replication and division during the late L1 stage (Hedgecock and White, 1985). The accumulation patterns of Worm-FUCCI were consistent with these observations. The presence of CDT-1^D^ only indicated that all of these cells arrested at the G1 phase during the first six hours of feeding of the starved L1 animals. Approximately 10 and 7 h after feeding, the int2 and int3 cells entered the S phase, as judged by the absence of CDT-1^D^. CDT-1^D^ was observed again roughly 12 h after feeding, indicating that the cells had entered the G2 phase. For the “int2D/V” cells, which undergo endoreplication without division, we referred to the relevant cell cycle phase as the G phase rather than the G2 phase due to the endoreplication without division. For the int3D/V cells, which undergo both DNA replication and division, we referred to the period of CDT-1^D^ re-accumulation before next round of division as G2 phase.

In summary, we generated Worm-FUCCI reporters whose accumulation dynamics faithfully indicates cell cycle progression in the Soma. Accumulation dynamics of the reporters during *C. elegans* embryogenesis demonstrated that only a few pairs of cells with an extended cell cycle length during the last round of division divide with a full cell cycle. The Worm-FUCCI strains constitute an invaluable resource for further study of coordination between cell cycle progression and cell fate differentiation, which has so far poorly defined in any species.

## Discussion

Embryonic cell cycles are unique in that barely any gap phase is present in the early embryo of most species, including *C. elegans* (Edgar and McGhee, 1988; Farrell and O’Farrell, 2014). However, the precise knowledge on gap phase introduction throughout embryogenesis remains elusive in all the metazoans studies so far. Here we developed Worm-FUCCI as faithful cell cycle progression reporters in *C. elegans* Soma. Automated profiling of the Worm-FUCCI accumulation allowed us to produce a first ever global picture of gap phase introduction throughout metazoan embryogenesis. These reporters greatly facilitate the future study of coordination between cell cycle progression and cell fate differentiation during development.

In *Drosophila melanogaster*, the first 13 embryonic cell cycles before mid-blastula transition (MBT) are synchronous but with increasing cell cycle length, which was thought to be dominated by the DNA replication checkpoint (Blythe and Wieschaus, 2015). After MBT, cell cycle length is mainly dictated by the introduction of gap phase which coincides with zygotic genome activation. Unlike *Drosophila* embryogenesis, cell division in *C. elegans* is asynchronous during the first cell division. Division asymmetry in cell cycle length between sister cells becomes increasingly frequent over development. Our data suggest that the overall cell cycle length and the division asymmetries in cell cycle length are mainly dictated by the S phase duration in most cells, although a short G1 phase that is beyond of detection by our reporter could also contribute to the asymmetry. Consistent with this, CDK-4 and Cyclin D (CYD-1), which are required for the progression through G1 phase during larval muscle development (Korzelius et al., 2011), are dispensable for most embryonic divisions except the following cases, i.e., the final division of some intestinal cells, the division of coelomocyte mother cells and the division of V5Q cells (Kipreos et al., 1996; Bao et al., 2008; Budirahardja and Gonczy, 2008; Clijsters et al., 2013; Adikes et al., 2020). Division asymmetry of the first cell cycle in *C. elegans* involves cell cycle check point protein and asymmetric regulation of DNA replication (Brauchle et al., 2003; Benkemoun et al., 2014).

The rapid and efficient degradation of the PIP-box-containing degron, CDT-1^D^, in the S phase makes this reporter an effective marker in accurately defining the boundary between the G1 and S phases. However, the Worm-FUCCI does have difficult in defining boundary between the S and G2 phase. Despite the lack of an obvious Cy motif in CDT-1, the degradation pathway of PIP box is conserved between worm and human. However, same as the degradation of PIP-box-containing CDT1 in human, the CDT-1^D^ of this study accumulates slowly from the G2 phase and peaks again in the G1 phase (Figures 1C, 3C), making it difficult in defining the boundary between S and G2 than that between G1 and S. The dynamics of CYB-1^D^ allow it to serve as a complement to the CDT-1^D^ in demarcating these boundaries. This is because that CYB-1^D^ shows no accumulation in the G1 phase when the abundance of CDT-1^D^ reaches its highest level; it starts to accumulate from the S phase and peaks in the G2 phase (Figures 3C, 4B). Therefore, the high level of CDT-1^D^ coupled with the absence of CYB-1^D^ is a reliable indicator of the G1 phase, and the absence of CDT-1^D^ coupled with a relatively low level of CYB-1^D^ is indicative of the S phase. Given that histone expression can serve as a clear indicator for the M phase, simultaneous accumulation of both reporters in the same cell can reliably indicate the G2 phase (Figure 1D). Apparently, our CDT-1^D^-based degradation cannot distinguish the G1 from G0 phase. Fortunately, a CDK sensor has recently been developed. The sensor consists of a fluorescently tagged CDK substrate that can steadily translocate from the nucleus to the cytoplasm in response to increasing CDK activity and consequent sensor phosphorylation, which enables distinguishing cycling cells in G1 from quiescent cells in G0 in *C. elegans* (Adikes et al., 2020).

Broad accumulation of the degron reporters was spatially and temporally achieved by *his-72* promoter and *pie-1* 3′UTR, providing an advantage in systematic delineation of cell cycle progression. Nevertheless, tissue-specific or conditionally expressed reporters may be necessary in other cases to facilitate the study of cell cycle progression and fate differentiation in specific cell types, especially during postembryonic development. Another limitation of the Worm-FUCCI reporter is the temporal sensitivity. Although the degradation of CDT-1^D^ is rapid, the degradation of CYB-1^D^ takes about 50 min to reach the basal level (Figures 3C, 4B). Also, the CDT-1^D^ accumulation is not fast enough to be detected due to a relatively slow maturation rate of mCherry, which may make a brief G1 phase undetectable. For example, only the two embryonic cells, “V5QL” and “V5QR,” were found to divide with a full cycle, which carry an apparent G1 phase. It remains possible that a brief gap phase with a very short duration, for example, within 10–20 min, is likely to be missed by our reporters. Consistent with this, depletion of *cyd-1* that is required for cell cycle progression through G1 phase not only led to abolishment of the last round of division, but also produced a sharp increase of CDT-1^D^ accumulation in the precursors of coelomocyte Supplementary Figure S8C,D), arguing the presence of a brief G1 phase that was missed by the reporter in the wild-type embryo. A robust quantification of Worm-FUCCI accumulation may help alleviate the problem but this is further complicated by a relatively low expression level derived from single-copy transgene. Therefore, a FUCCI reporter with a faster maturation time than the mCherry is needed to provide a better temporal resolution in deducing a short G1 phase. For example, mRFP1 is approximately 15 min faster than mCherry (Balleza et al., 2018). mNeonGreen, another fast-folding fluorescence protein has a maturation time less than 10 min (Hirano et al., 2022). However, the photobleaching and quantum yield of fluorescence protein have to be taken into consideration in prioritizing a reporter. Despite the broad expression of the reporters, we observed a slightly lower expression level of CDT-1^D^ in some sublineages, including a few cells in the D sublineages. However, further curation of the cell lineage with an extended time point revealed a clear CDT-1^D^ accumulation in those D sublineage cells (Supplementary Figure S1). The lack of CDT-1^D^ accumulation in the Z2 and Z2 cells (Supplementary Figure S1) were probably due to maternal inhibition or transcriptional quiescence (Ghosh and Seydoux, 2008; Guven-Ozkan et al., 2008), or the inability of the *his-72* promoter in driving zygotic expression, making it unsuitable for monitoring cell cycle progression of the two germline progenitor cells.

The broad accumulation of the Worm-FUCCI offers an opportunity to study the coordination of the cell cycle and cell fate differentiation during postembryonic development, including development of seam cells and vulva. Given that some of the postembryonic intestine cells are known to undergo endoreplication, the dynamics of the reporters in these cells also lay a foundation for mechanistic research into the regulation of endoreplication. However, the CYB-1-based reporter may not be suitable for deducing cell cycle progression in the germline due to their discordant of accumulation dynamics (Supplementary Figures S10–12).

## Data Availability

The original contributions presented in the study are included in the article/Supplementary Material, further inquiries can be directed to the corresponding author.
