# Supplementary material for: Initial characterization of gap phase introduction in every cell cycle of C. elegans embryogenesis
Source: Front Cell Dev Biol. 2022 Oct 25;10:978962. doi: 10.3389/fcell.2022.978962 (PMC9641140; doi:10.3389/fcell.2022.978962)
Supplement: Supplementary file 1 [file DataSheet2.PDF]

Strain Name	Genotype	Phenotype
RW10029	unc-119(ed3) III; zuIs178 [his-72(1kb 5' UTR)::his-72::SRPVAT::GFP::his-72 (1KB 3' UTR) + 5.7 kb XbaI - HindIII unc-119(+)]; stIs10024 [pie-1::H2B::GFP::pie-1 3' UTR + unc-119(+)]	GFP lineaging marker
RW10226	unc-119(ed3) III; stIs10226 [Phis-72::HIS-24::mCherry ::let-858 3' UTR + unc-119(+)]; stIs37[Ppie-1::mCherry::H2B::pie-1 3'UTR + unc-119(+)]	RFP lineaging marker
ZZY0576	unc-119(tm4063) III; zzyIs176 [Phis-72::mCherry::CDT-1(1-189 aa)::pie-1 3'UTR + unc-119(+)] V	FUCCI(mCherry, maternal and zygotic)
ZZY0578	unc-119(tm4063) III; zzyIs176 [Phis-72::mCherry::CDT-1(1-189 aa)::pie-1 3'UTR + unc-119(+)] IV	FUCCI(mCherry, zygotic only)
ZZY0536	unc-119(tm4063) III; zzyIs176 [Phis-72::mCherry::CDT-1(1-189 aa)::pie-1 3'UTR + unc-119(+)] V; unc-119(ed3) III; zuIs178 [his-72(1kb 5' UTR)::his-72::SRPVAT::GFP::his-72 (1KB 3' UTR) + 5.7 kb XbaI - HindIII unc-119(+)]; stIs10024 [pie-1::H2B::GFP::pie-1 3' UTR + unc-119(+)]	GFP lineaging marker, FUCCI(mCherry, maternal and zygotic)
ZZY0538	unc-119(tm4063) III; zzyIs176 [Phis-72::mCherry::CDT-1(1-189 aa)::pie-1 3'UTR + unc-119(+)] II; unc-119(ed3) III; zuIs178 [his-72(1kb 5' UTR)::his-72::SRPVAT::GFP::his-72 (1KB 3' UTR) + 5.7 kb XbaI - HindIII unc-119(+)]; stIs10024 [pie-1::H2B::GFP::pie-1 3' UTR + unc-119(+)]	GFP lineaging marker, FUCCI(mCherry, zygotic only)
ZZY0678	unc-119(tm4063) III; zzyIs147 [Phis-72::mCherry::EGL-13(1-25 aa)::CYB-1(8-80 aa)::pie-1 3'UTR + unc-119(+)] III	FUCCI(mCherry, maternal and zygotic)
ZZY0686	unc-119(tm4063) III; zzyIs147 [Phis-72::mCherry::EGL-13(1-25 aa)::CYB-1(8-80 aa)::pie-1 3'UTR + unc-119(+)] III; unc-119(ed3) III; zuIs178 [his-72(1kb 5' UTR)::his-72::SRPVAT::GFP::his-72 (1KB 3' UTR) + 5.7 kb XbaI - HindIII unc-119(+)]; stIs10024 [pie-1::H2B::GFP::pie-1 3' UTR + unc-119(+)]	GFP lineaging marker, FUCCI(mCherry, maternal and zygotic)
ZZY0717	unc-119(tm4063) III; zzyIs180 [Phis-72::GFP::EGL-13(1-25 aa)::CYB-1(8-80 aa)::pie-1 3'UTR + unc-119(+)] II	FUCCI(GFP, maternal and zygotic)
ZZY0731	unc-119(tm4063) III; zzyIs176 [Phis-72::mCherry::CDT-1(189 aa)::pie-1 3'UTR + unc-119(+)] V; zzyIs180 [Phis-72::GFP::EGL-13(1-25 aa)::CYB-1(8-80 aa)::pie-1 3'UTR + unc-119(+)] II	FUCCI(mCherry, GFP)
ZZY0732	unc-119(tm4063) III; zzyIs180 [Phis-72::GFP::EGL-13(1-25 aa)::CYB-1(8-80 aa)::pie-1 3'UTR + unc-119(+)] II; unc-119(ed3) III; stIs10226 [Phis-72::HIS-24::mCherry ::let-858 3' UTR + unc-119(+)]; stIs37[Ppie-1::mCherry::H2B::pie-1 3'UTR + unc-119(+)]	RFP lineaging marker, FUCCI(GFP, maternal and zygotic)
