# Supplementary figures and images for: Initial characterization of gap phase introduction in every cell cycle of C. elegans embryogenesis
Source: Front Cell Dev Biol. 2022 Oct 25;10:978962. doi: 10.3389/fcell.2022.978962 (PMC9641140; doi:10.3389/fcell.2022.978962)

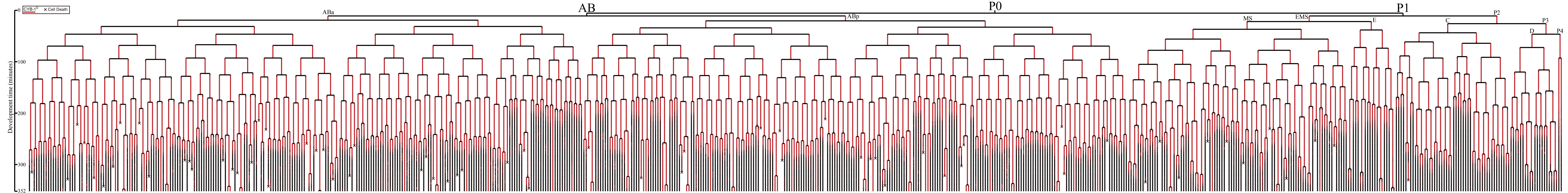

Supplement: Supplementary file 3 [file Image2.pdf]

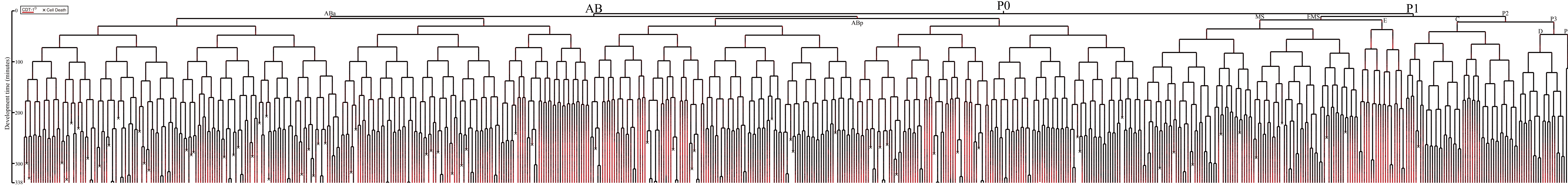

Supplement: Supplementary file 7 [file Image1.pdf]
