# Supplementary material for: Initial characterization of gap phase introduction in every cell cycle of C. elegans embryogenesis
Source: Front Cell Dev Biol. 2022 Oct 25;10:978962. doi: 10.3389/fcell.2022.978962 (PMC9641140; doi:10.3389/fcell.2022.978962)
Supplement: Supplementary file 4 [file DataSheet3.PDF]

**Fig. S1** Lineal accumulation dynamics of mCherry::CDT-1 (CDT-1$^{D}$) from four-cell to comma stage of a *C. elegans* embryo. Cell deaths are indicated with a "×". (See figure at the bottom)

**Fig. S2** Lineal accumulation dynamics of mCherry::CYB-1 (CYB-1$^{D}$) from four-cell to comma stage of a *C. elegans* embryo. Cell deaths are indicated with a "×". (See figure at the bottom)

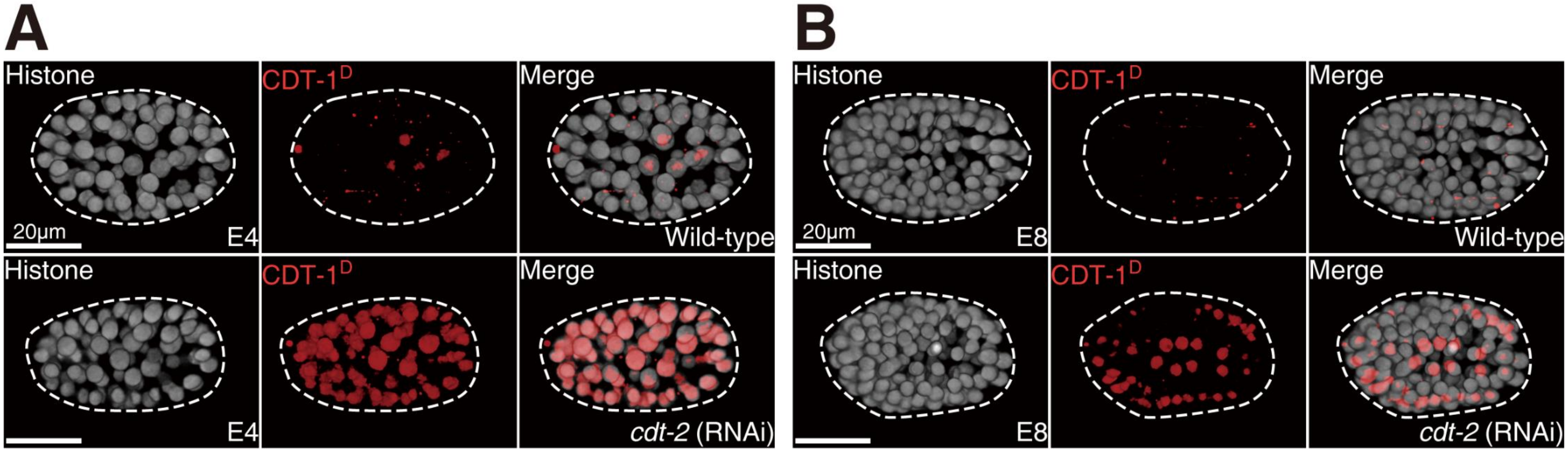


**Fig. S3** Validation of CDT-1$^{D}$ degradation by RNAi against *cdt-2*, a known component of CDT-1 degradation pathway.

A. Shown are epifluorescence micrographs of embryonic accumulation of CDT-1$^{D}$ (red) or Histone::GFP (grey) in the wild-type (top) and the *cdt-2* RNAi (bottom) animals. Micrographs are taken at comparable E4 (E produced into 4 progeny) stage. Note that after *cdt-2* perturbation, CDT-1 accumulation was retained in most nuclei. Histone, HIS-72::GFP/H2B::GFP used for cell tracking.
B. Same as "A" except the micrographs were acquired at E8 stage (E produced 8 progeny).

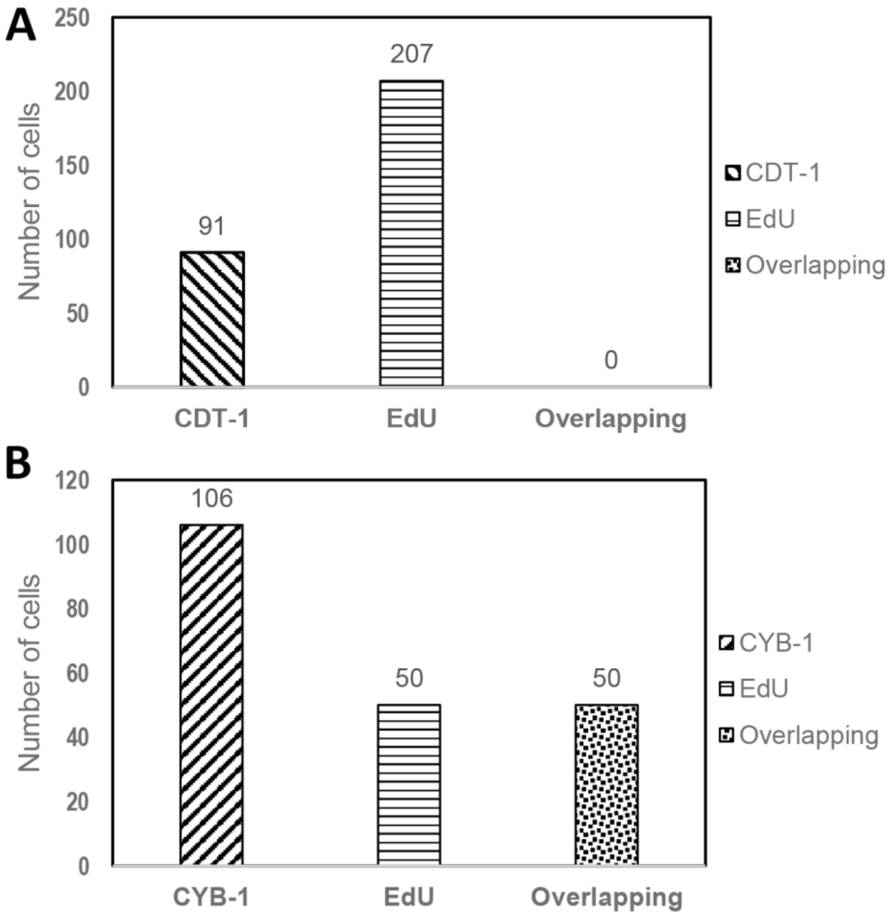


**Fig. S4** Quantification of overlapping cells that are both EdU positive and CDT-1$^{D}$ (A) or CYB-1$^{D}$ positive (B).

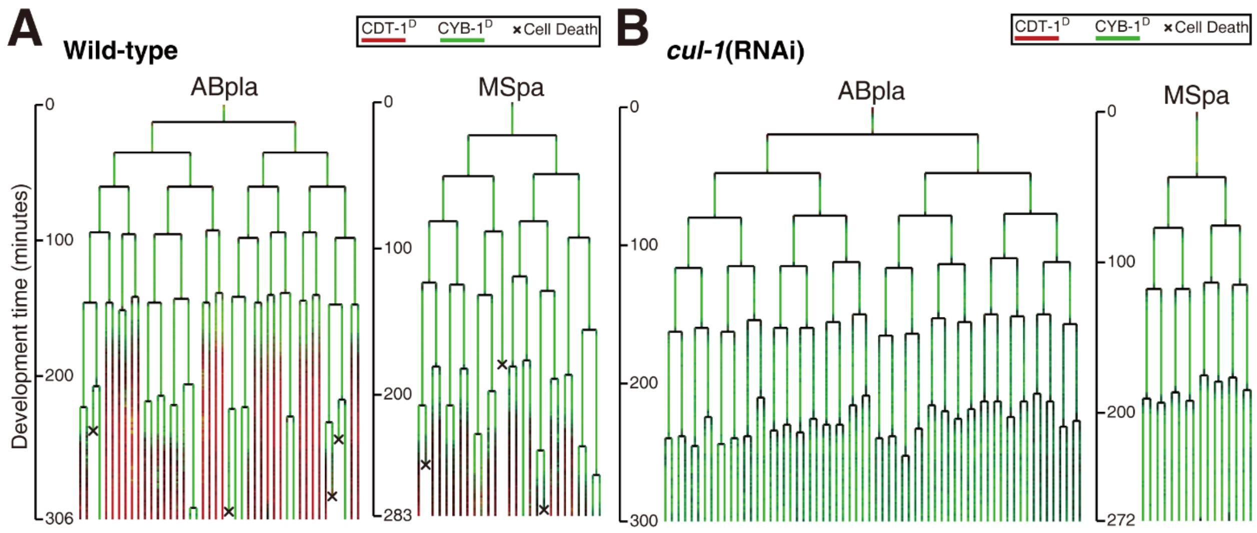


**Fig. S5** Changes in the accumulation dynamics of Worm-FUCCI are indicative of cell cycle exit.

A. Superimposed lineal accumulation of CDT-1$^{D}$ (red) and CYB-1$^{D}$ (green) in the representative cell sublineages, "ABpla" and "MSpa", in the wild-type (N2) embryo. Development time starting from the birth of the cell of interest is shown on the left and cell death is indicated with a "×".

B. Same as "A" except the embryos are retrieved from the parents treated by RNAi against *cul-1*. Each cell lineage tree is shown up to the developmental time similar to that in the wild-type embryo. Note that the absence of CDT-1$^{D}$ but with apparent CYB-1$^{D}$ accumulation is indicative of the failure of cell cycle exit in the RNAi embryo.

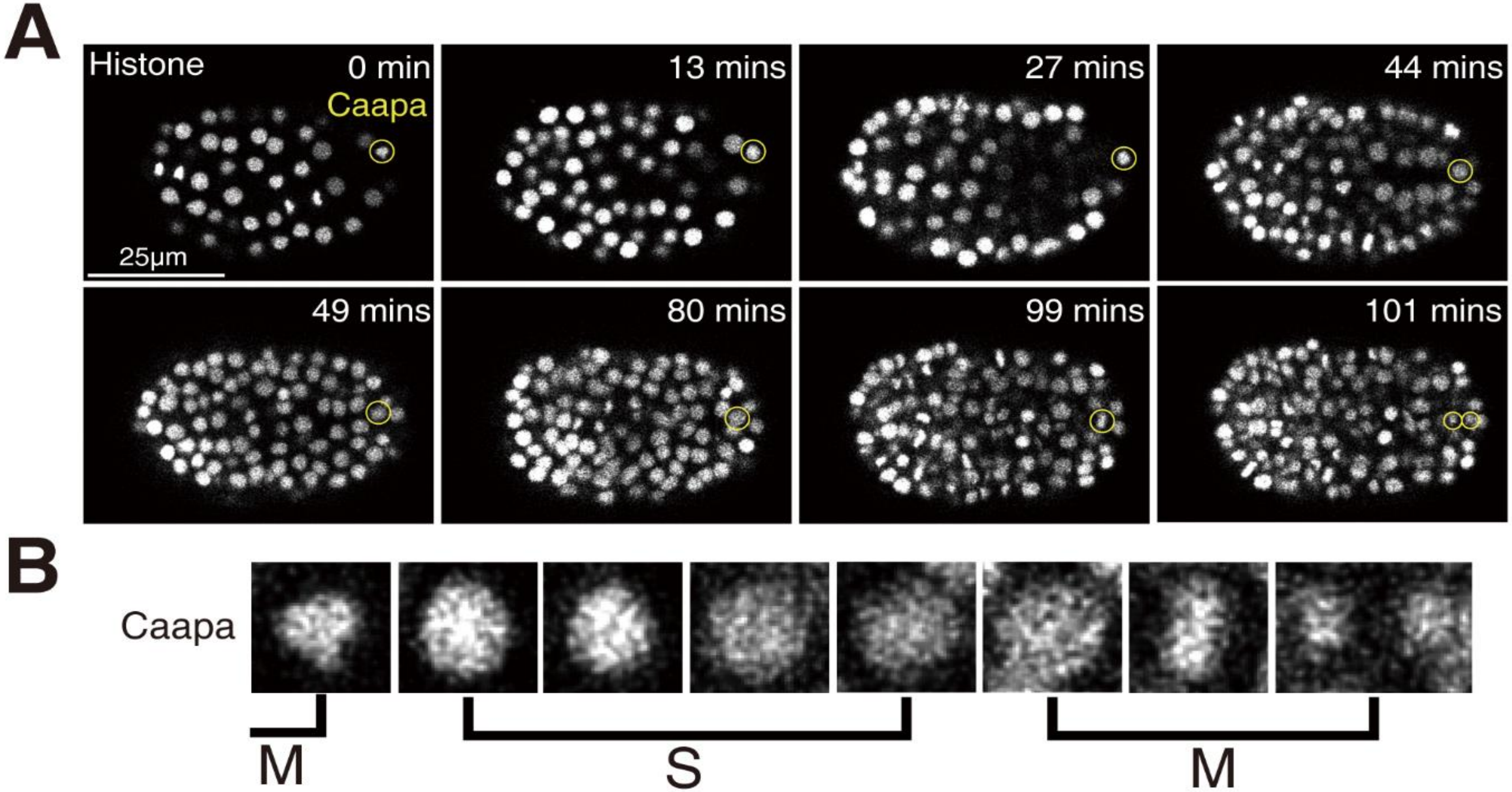


**Fig. S6** Histone accumulation over the cell cycle of "Caapa".

A. Shown are epifluorescence micrographs of histone::GFP (gray) taken at eight time points starting from the birth of "Caapa" cell. The cell and its daughters are highlighted with yellow circles. Note that cell with condensed chromatin divides within two minutes, indicated as 99 to 101 minutes.

B. Magnified views of histone::GFP in the developing "Caapa" cell and its daughters as shown in "A". Note that the cell initiates mitosis two minutes after chromatin condensation, indicating that its elongated cell cycle duration is due to its extended S phase rather than M phase.

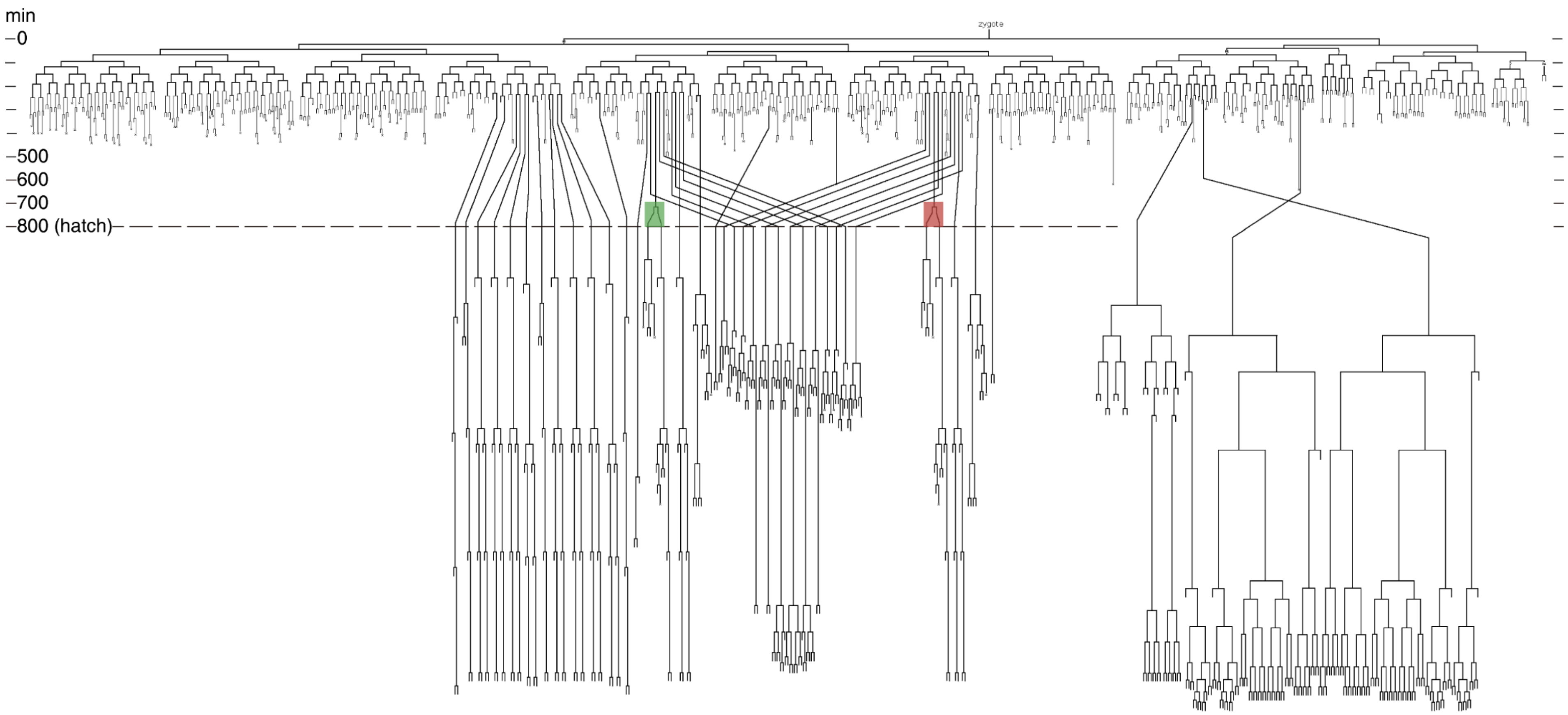


**Fig. S7** Cells "ABplapapaa" and "ABprapapaa" shown in the context of a complete cell lineage tree of *C. elegans* embryo.

The divisions of the two cells are shaded in green and red, respectively (modified from Sulston et al., (1983)[15]). The two cells divided roughly one hour before hatching. Development time (minute) at 20 ºC after fertilization is shown on the left. Original image by J .E. Sulston, E. Schierenberg, J. G. White, J. N. Thomson. Modified with permission from WormAtlas.

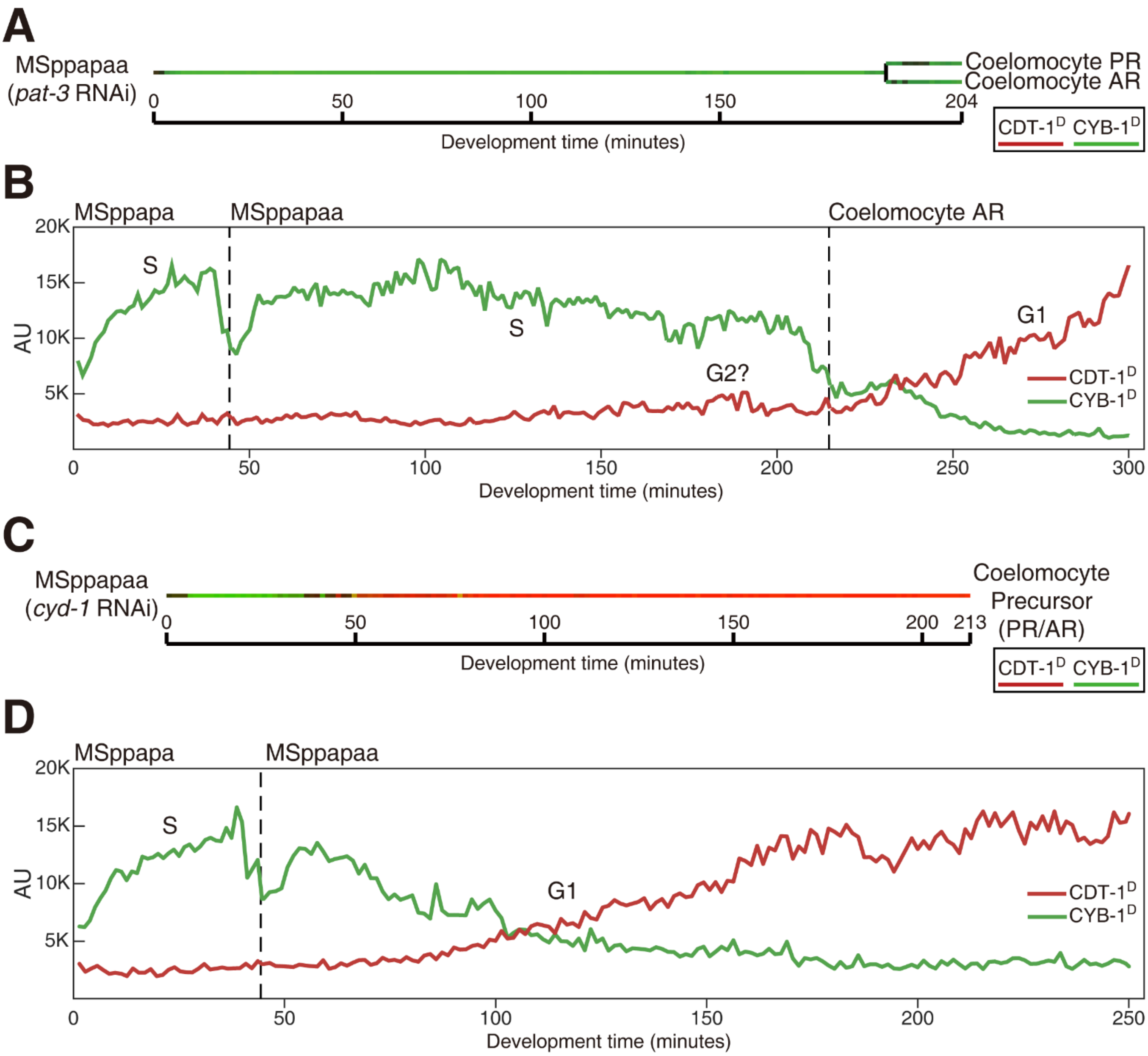


**Fig. S8** Worm-FUCCI accumulation during cell cycle progression of coelomocyte precursor "MSppapaa".

A. Superimposed lineal accumulation of CDT-1$^{D}$ (red) and CYB-1$^{D}$ (green) in one of the coelomocytes and their precursor (MSppapaa) in the embryos treated by RNAi against *pat-3*. Developmental time in minute starting from the birth of the precursors is shown underneath the lineal accumulation tree.
B. Quantification of the accumulation of CDT-1$^{D}$ (red) and CYB-1$^{D}$ (green) in the coelomocyte AR and its ancestors. Normalized fluorescence intensity in arbitrary unit (AU) is plotted on the Y axis, and development time of MSppapa into coelomocyte AR from its birth on the X axis. Cell cycle phases are indicated based on the accumulation dynamics of the two reporters. Division time points are indicated with dashed line.
C. Same as "A" except the embryos treated by the RNAi against *cyd-1*. Note the division was abolished and CDT-1$^{D}$ initiated accumulation.

D. Same as “B” except the embryos treated by the RNAi against *cyd-1*. Note the apparent and marginal accumulations of the CDT-$1^{D}$ in the cell cycle of “MSppapaa” in the RNAi and wild-type embryos, respectively.

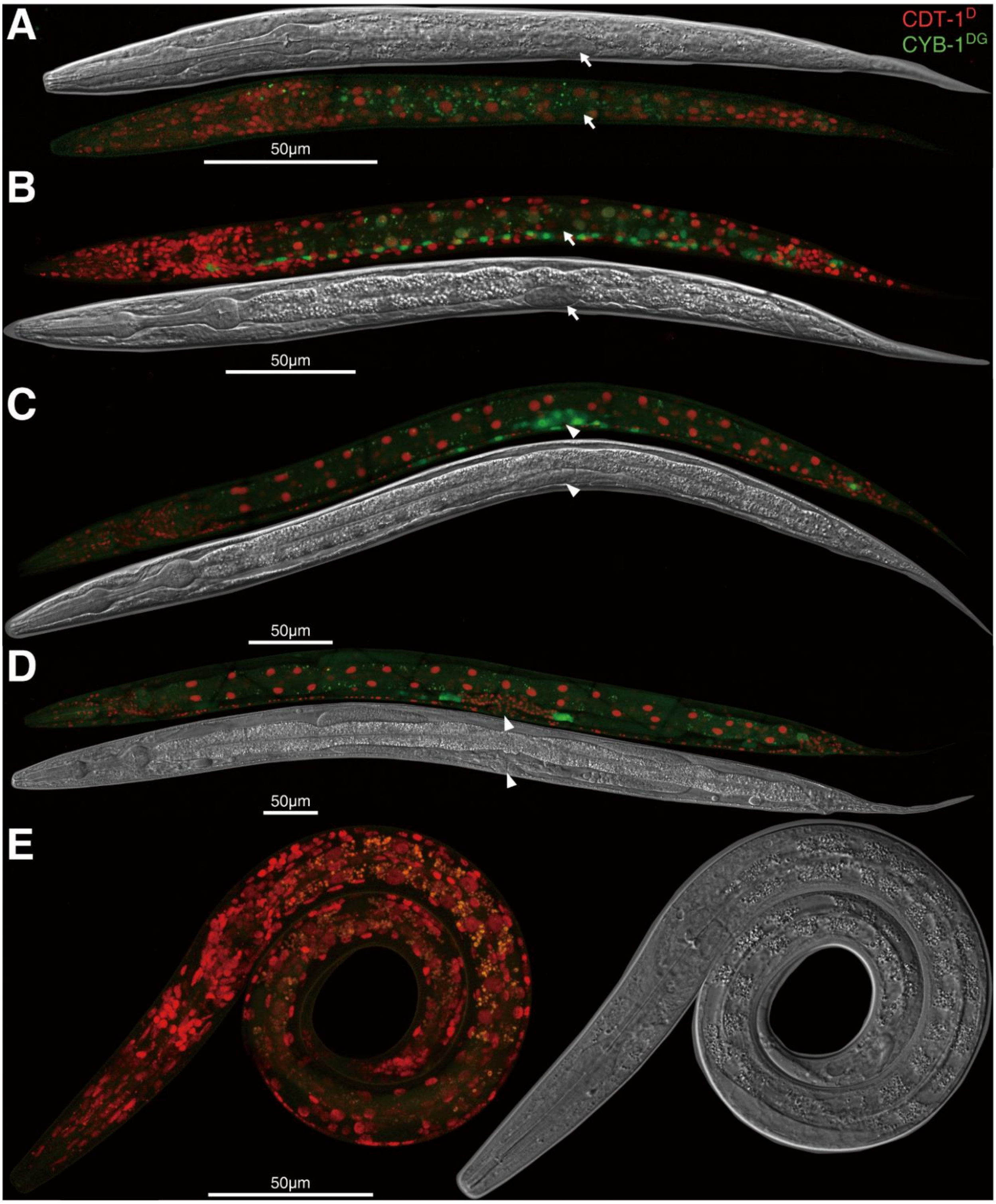


**Fig. S9** Accumulation of Worm-FUCCI in larvae.

Shown are 3D projections of superimposed accumulations of CDT-1$^{D}$ (red) and CYB-1$^{DG}$ (green) in L1 (A), L2 (B), L3 (C), L4 (D) and dauer larvae (E). Nomarski micrographs of the same animals are also shown. Developing gonads and vulva are indicated with arrow and arrowhead, respectively.

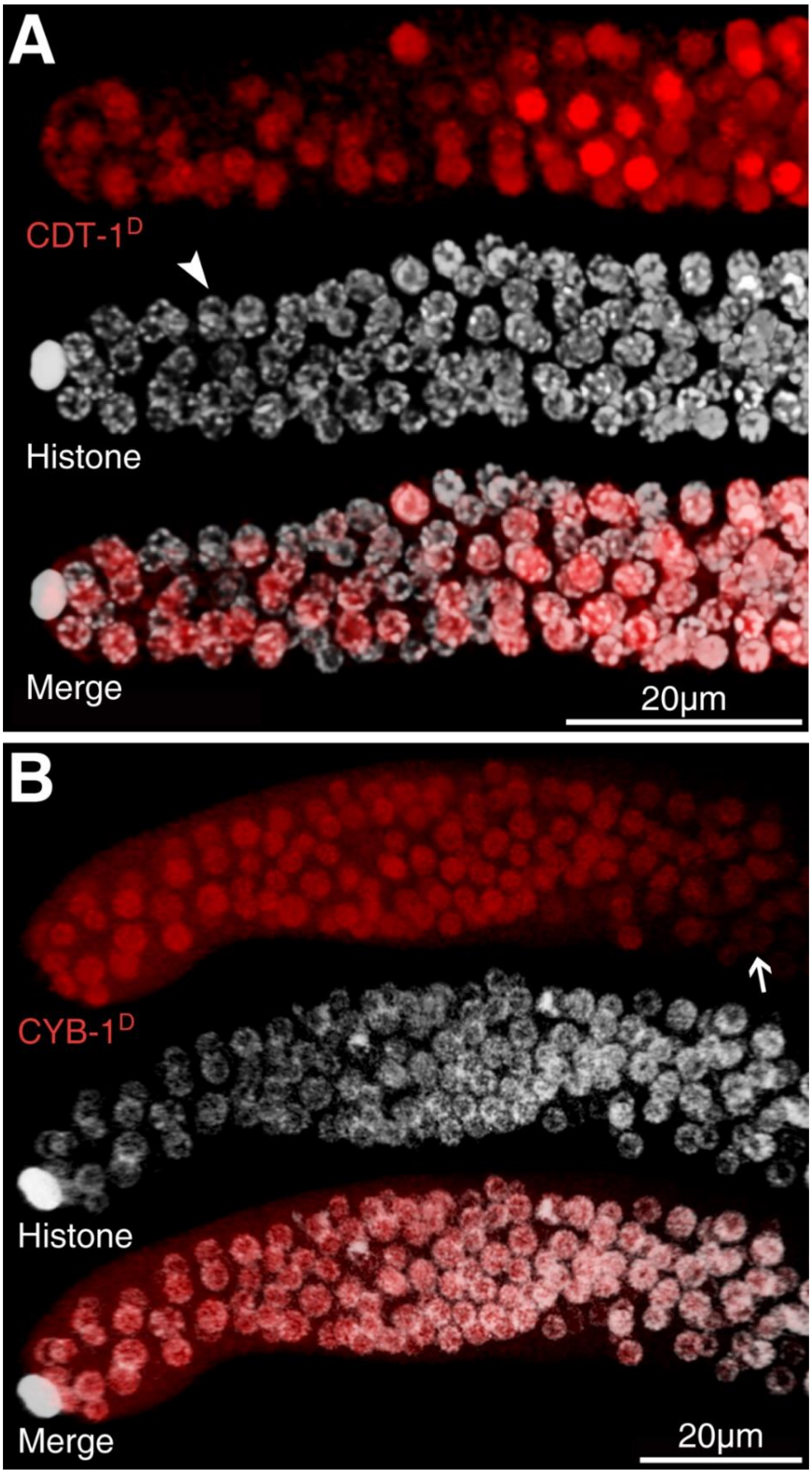


**Fig. S10** Accumulation dynamics of Worm-FUCCI in the mitotic germline.

A. Accumulation dynamics of CDT-1$^{D}$ in the mitotic zone. Shown are epifluorescence micrographs of CDT-1$^{D}$ (red, top), histone::GFP (grey, middle) and merge between the two (bottom). A representative nucleus without CDT-1$^{D}$ accumulation is indicated by an arrowhead.

B. Accumulation dynamics of CYB-1$^{D}$ in the mitotic zone. Shown are epifluorescence micrographs of CYB-1$^{D}$ (red, top), histone::GFP (grey, middle) and merge between the two (bottom). A region with decreased accumulation of is indicated by arrow.

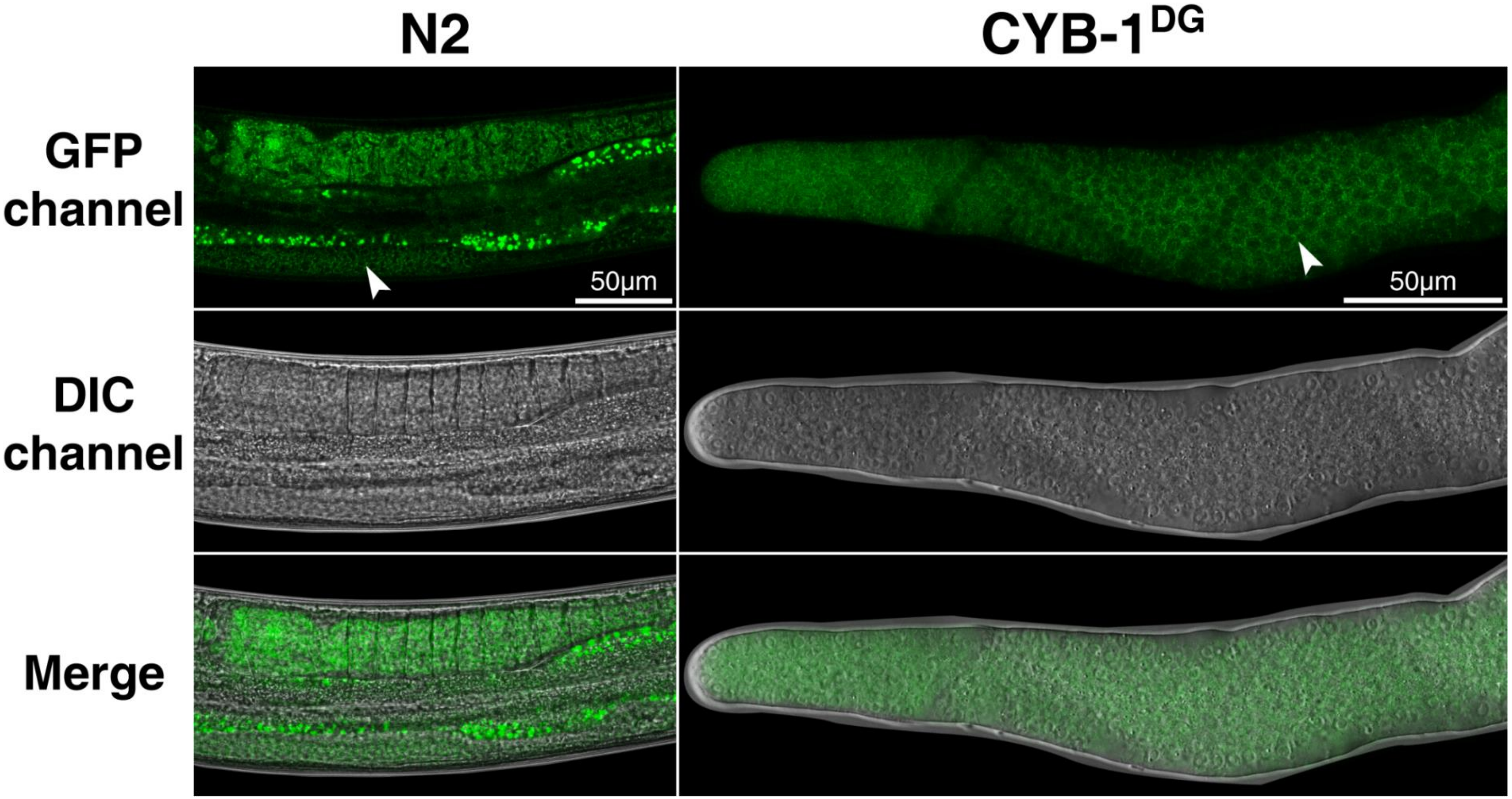


**Fig. S11** Autofluorescence of mitotic germline in *C. elegans*.

Left: Autofluorescence was detected in the mitotic zone of wild type (N2) germline in the context of young adult illuminated with blue light (488 nm) (indicated with arrowhead).

Right: Autofluorescence was detected in the mitotic zone of isolated germline (indicated with arrowhead) from young animal expressing CYB-1$^{DG}$ illuminated with blue light (488 nm).

Top: GFP. Middle: DIC. Bottom: Merge between the two above.

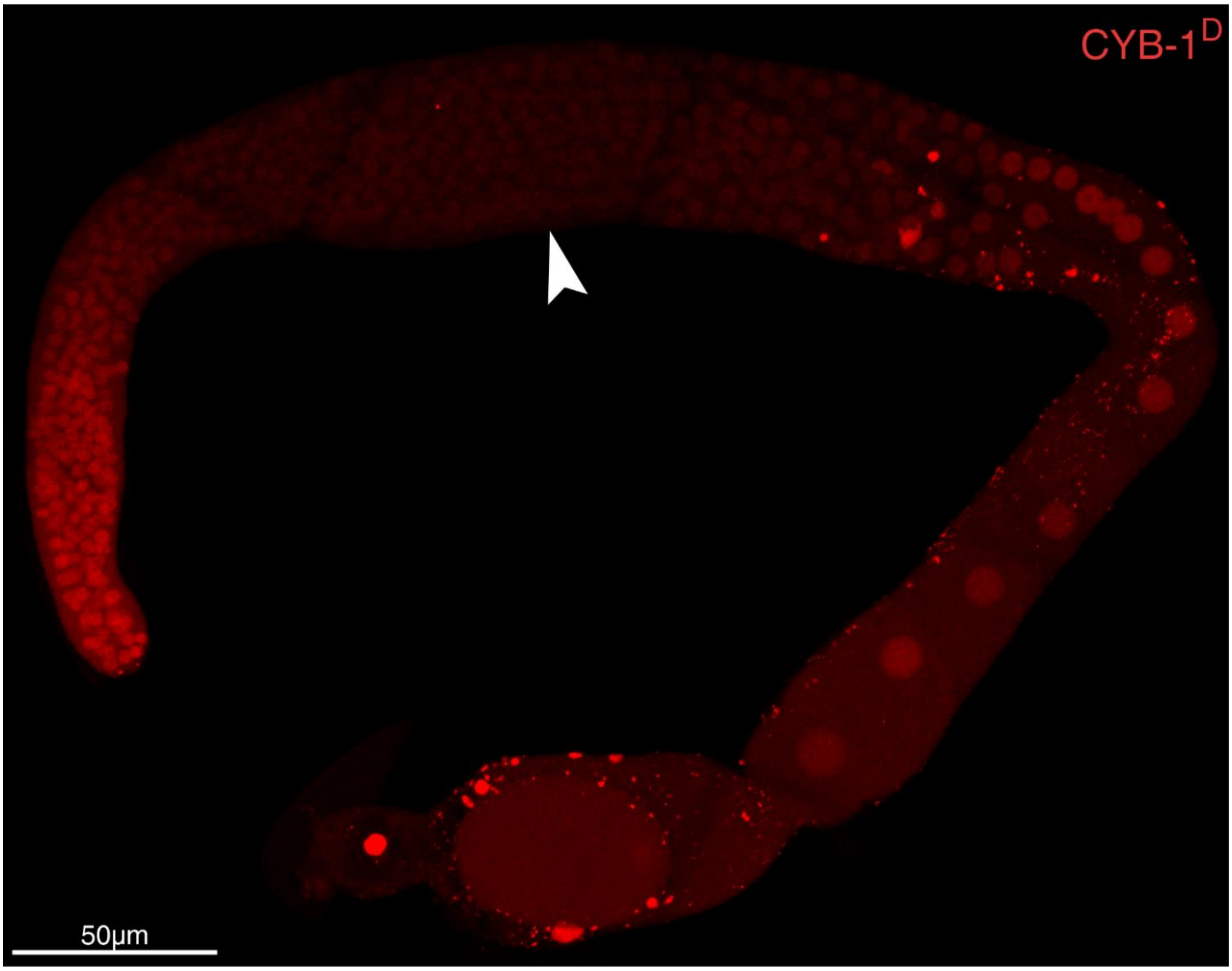


**Fig. S12** A global view of accumulation of CYB-1$^{D}$ in the germline (indicated with arrowhead).

Note a significant drop of fluorescence intensity in the cells at pachytene region compared with those in the mitotic zone.

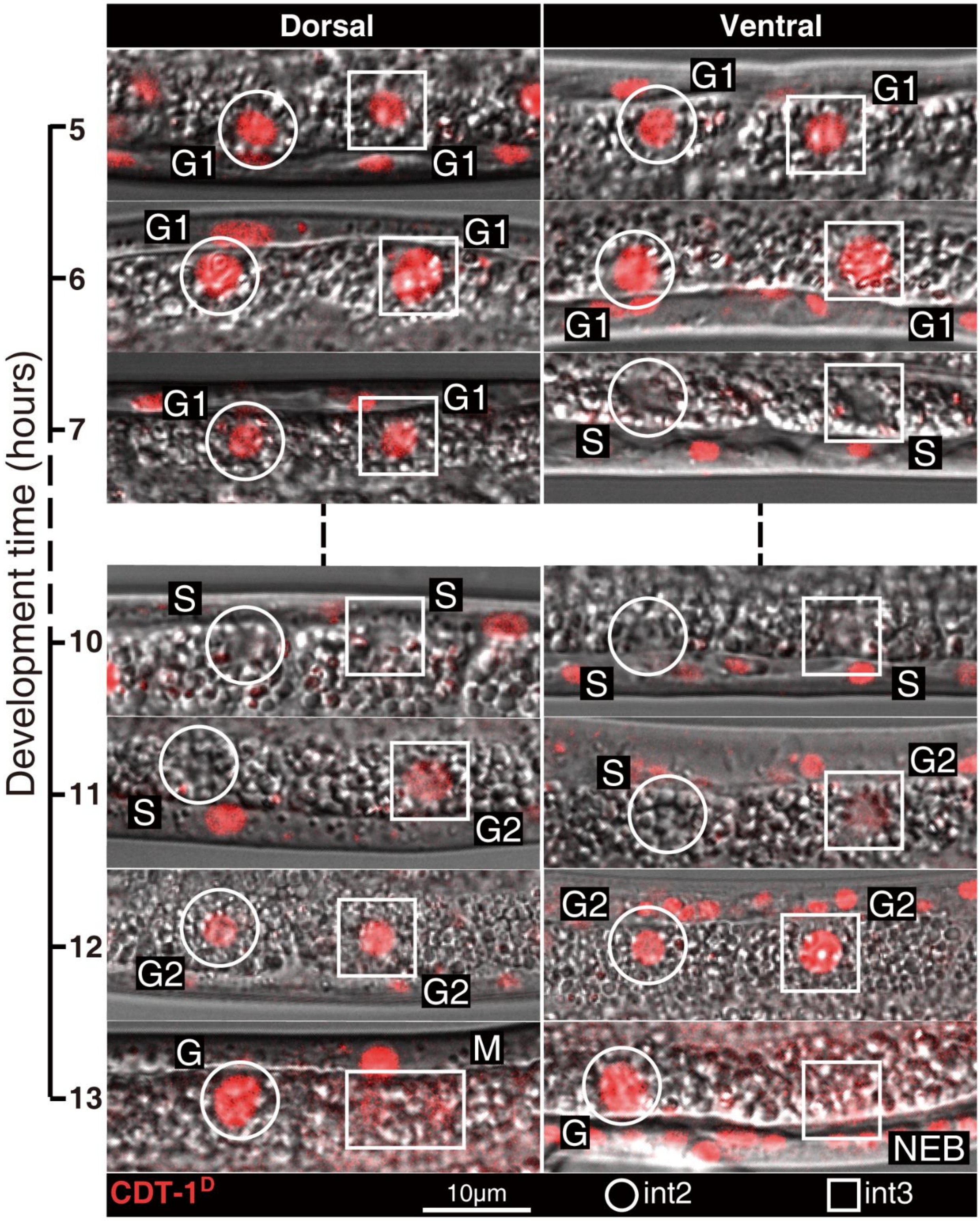


**Fig. S13** Accumulation dynamics of CDT-1$^{D}$ in the intestine cells "int2" (circled) and "int3" (squared) in developing L1 larvae.

Shown are the epifluorescence micrographs superimposed with corresponding Nomarski micrographs. The dorsal and ventral sides of cell pairs are shown on the left and right, respectively. Cell cycle phases revealed by CDT-1$^{D}$ are indicated. The cell cycle phase that cannot be defined as either G1 or G2 phase is labeled as G phase (G). Development time (hour) after feeding is indicated on the left (see MATERIALS AND METHODS).

**Table S1** List of worm strains and their genotypes used in this study.

**Table S2** List of plasmids and worm strains generated in this study.

## Supplemental Video legends

**Video. S1** Accumulation of Worm-FUCCI (CDT-$1^{D}$, CYB-$1^{DG}$) in a rotating embryo.

**Video. S2** Accumulation of the Worm-FUCCI from comma stage to two-fold stage in an embryo perturbed by RNAi against *pat-3*.
