# Supplementary material for: Initial characterization of gap phase introduction in every cell cycle of C. elegans embryogenesis
Source: Front Cell Dev Biol. 2022 Oct 25;10:978962. doi: 10.3389/fcell.2022.978962 (PMC9641140; doi:10.3389/fcell.2022.978962)
Supplement: Supplementary file 5 [file DataSheet1.PDF]

Table S1 List of worm strains and their genotypes used in this study.

Plasmid name	Genotype
pZZ141	A1::Phis-72::mCherry::HIS-24::pie-1 3'UTR+ unc-119(+)
pZZ176	A1::Phis-72::mCherry::CDT-1(189aa)::pie-1 3'UTR+ unc-119(+)
pZZ147	A1::Phis-72::mCherry::EGL-13(1-25aa)::CYB-1(8-80aa)::pie-1 3'UTR + unc-119(+)
pZZ180	A1::Phis-72::GFP::EGL-13(1-25aa)::CYB-1(8-80aa)::pie-1 3'UTR+ unc-119(+)
